# Whole‐Genome Approach Discovers Novel Genetic and Nongenetic Variance Components Modulated by Lifestyle for Cardiovascular Health

**DOI:** 10.1161/JAHA.119.015661

**Published:** 2020-04-20

**Authors:** Xuan Zhou, Julius van der Werf, Kristin Carson‐Chahhoud, Guiyan Ni, John McGrath, Elina Hyppönen, S. Hong Lee

**Affiliations:** ^1^ Australian Centre for Precision Health University of South Australia Adelaide South Australia Australia; ^2^ School of Environmental and Rural Science University of New England Armidale New South Wales Australia; ^3^ Institute for Molecular Bioscience University of Queensland Brisbane Queensland Australia; ^4^ Queensland Brain Institute University of Queensland Brisbane Queensland Australia; ^5^ Queensland Centre for Mental Health Research The Park Centre for Mental Health Wacol Queensland Australia; ^6^ South Australian Health and Medical Research Institute Adelaide South Australia Australia

**Keywords:** cardiovascular traits, genotype–lifestyle interaction, lifestyle, residual–lifestyle interaction, whole‐genome approach, Genetic, Association Studies, Genetics, Basic Science Research

## Abstract

**Background:**

Both genetic and nongenetic factors can predispose individuals to cardiovascular risk. Finding ways to alter these predispositions is important for cardiovascular disease prevention.

**Methods and Results:**

We used a novel whole‐genome approach to estimate the genetic and nongenetic effects on—and hence their predispositions to—cardiovascular risk and determined whether they vary with respect to lifestyle factors such as physical activity, smoking, alcohol consumption, and dietary intake. We performed analyses on the ARIC (Atherosclerosis Risk in Communities) Study (N=6896–7180) and validated findings using the UKBB (UK Biobank, N=14 076–34 538). Lifestyle modulation was evident for many cardiovascular traits such as body mass index and resting heart rate. For example, alcohol consumption modulated both genetic and nongenetic effects on body mass index, whereas smoking modulated nongenetic effects on heart rate, pulse pressure, and white blood cell count. We also stratified individuals according to estimated genetic and nongenetic effects that are modulated by lifestyle factors and showed distinct phenotype–lifestyle relationships across the stratified groups. Finally, we showed that neglecting lifestyle modulations of cardiovascular traits would on average reduce single nucleotide polymorphism heritability estimates of these traits by a small yet significant amount, primarily owing to the overestimation of residual variance.

**Conclusions:**

Lifestyle changes are relevant to cardiovascular disease prevention. Individual differences in the genetic and nongenetic effects that are modulated by lifestyle factors, as shown by the stratified group analyses, implies a need for personalized lifestyle interventions. In addition, single nucleotide polymorphism–based heritability of cardiovascular traits without accounting for lifestyle modulations could be underestimated.

**Clinical Perspective**

**What Is New?**

- A novel whole‐genome approach reveals that lifestyle factors can modulate genetic and nongenetic effects on cardiovascular traits.

**What Are the Clinical Implications?**

- Lifestyle changes are relevant to cardiovascular disease prevention, but the associated cardiovascular health benefits are greater for some individuals than others, implying a need for personalized lifestyle interventions.

**Nonstandard Abbreviations and Acronyms**

ARIC Atherosclerosis Risk in Communities

G‐C genotype–covariate

GREML genomic restricted maximum likelihood

MRNM multivariate reaction norm model

R‐C residual–covariate

RNM reaction norm model

UKBB UK Biobank

Cardiovascular diseases (CVDs) are the world's number 1 cause of mortality, claiming an estimated total of 17.9 million lives globally in the year 2016 alone—that is 31% of the total deaths in a single year.^1^ Managing CVD risk is therefore a top public health priority worldwide. It is estimated that between 20% and 60% phenotypic variability in CVD‐related traits such as blood pressure and blood‐clotting factors are attributed to additive genetic variation (see ref.^2^, ^3^, ^4^, ^5^, ^6^), and the remaining 40% to 80%, commonly referred to as residual variation, could arise from random measurement errors and systematic nongenetic variation in the epigenome, transcriptome, metabolome, proteome, and microbiome, which are involved in or interact with the translation of genotype to phenotype.

Given the substantial genetic and nongenetic contributions to CVD risk, identifying ways that modify their effects can have important implications for CVD prevention. In fact, the idea of genotype–environment or genotype–covariate (G‐C) interaction is well established for traits such as body mass index (BMI).^7^, ^8^, ^9^ That is, genetic effects vary depending on environmental exposure, such as modifiable lifestyle covariates including smoking, alcohol intake, and physical activity. Similar to G‐C interaction to genetic variance, we recently demonstrated that some nongenetic variance components can exist that change with respect to lifestyle covariates, which we termed residual–covariate (R‐C) interaction,^10^ that is, phenotypic variation with respect to lifestyle covariates that is independent of genetic effects.

Understanding G‐C and R‐C interactions in the context of cardiovascular traits will not only translate into empowering public messages but also enable personalized lifestyle changes for CVD prevention according to individuals’ genetic and nongenetic information, as opposed to a one‐fits‐all approach that neglects individual differences. Aside from its practical implications, studying G‐C and R‐C interactions is also of theoretical value as it may offer some insight into missing heritability.^11^, ^12^

To date, G‐C interaction estimates for cardiovascular traits are based on a limited number of genetic variants^13^, ^14^, ^15^, ^16^, ^17^, ^18^, ^19^, ^20^; therefore they are likely underestimated. R‐C interaction has been largely neglected, leading to potential confounding between G‐C and R‐C interactions in the presence of genuine R‐C interaction.^10^ In this article, using a novel whole‐genome approach,^10^ we extend the current understanding of G‐C and R‐C interactions on cardiovascular health. Instead of focusing on a few genetic variants with large phenotypic effects, our approach uses all common single nucleotide polymorphisms (SNPs) capturing variation across the entire genome, thereby providing genome‐wide estimates of G‐C interaction. Furthermore, our approach allows residual variance to vary with respect to a chosen covariate, thereby providing estimates of R‐C interaction. By examining G‐C and R‐C interactions, we identify lifestyle factors that modulate genetic and/or nongenetic effects on traits that are indicative of CVD risk.

## Methods

Simulated data used in this article can be obtained from the authors upon request. Our access to the ARIC (Atherosclerosis Risk in Communities) Study data was under the code phs000090, and access to the UKBB (UK Biobank) data was approved by the UKBB research ethics committee under the reference number 14 575.

Our analyses were based on the following 2 data sets: the ARIC Study and the UKBB. Because of the sensitive nature of the data collected for this study, requests to access the data sets from qualified researchers may be sent to the database of Genotypes and Phenotypes (dbgap-help@ncbi.nlm.nih.gov) and the UKBB (access@ukbiobank.ac.uk). The ARIC Study was chosen for our primary analyses because it covers a wider range of cardiovascular traits than the latter data set. The UKBB, which has a larger sample size than the ARIC Study, was chosen as the validation set. The sample sizes for our analyses depended on the availability of phenotype and genotype data, which varied between studies and cross traits. For the ARIC Study, the sample sizes were between 6896 and 7180. For the UKBB, the sample sizes were between 14 076 and 34 538.

### Ethics Statement

The current study was approved by the University of South Australia Human Research Ethics Committee. The ARIC Study was approved by the institutional review boards of all participating institutions, including the University of Minnesota, Johns Hopkins University, University of North Carolina, University of Mississippi Medical Centre, and Wake Forest University. The UKBB was approved by the North West Multi‐centre Research Ethics Committee (11/NW/0382). All ARIC Study and UKBB participants gave written informed consent.

### ARIC Study

The ARIC Study is a prospective study on the cause of atherosclerosis, with data collected from up to 5 visits over 15 years from participants of 4 U.S. communities (Forsyth County, NC; Jackson, MS; suburbs of Minneapolis, MN; and Washington County, MD) who were aged between 45 and 64 years in 1987 to 1989.^21^ To maximize the sample size for our analyses, we only used data from visit 1 that occurred from 1987 to 1989.

#### Cardiovascular Traits

A total of 23 cardiovascular health‐related traits were selected. Coagulation factors were determined in the ARIC Central Hemostasis Laboratory using previously published procedures.^22^ Plasma concentration of fibrinogen was measured by the thrombin–titration method, factor VII and factor VIII activity by clotting assays, and Von Willebrand's factor antigen with an ELISA technique.^23^, ^24^ P‐R interval, Q‐T interval, QRS interval, and Cornell voltage were derived from standard 12‐lead electrocardiography.^25^, ^26^ Sitting blood pressure (systolic and diastolic) was measured 3 times from the right arm and calculated using the average of the last 2 readings. Pulse pressure was computed as the difference between systolic and diastolic blood pressure.

#### Lifestyle Covariates

A total of 22 lifestyle covariates were selected. Smoking was indexed by “cigarette years of smoking,” derived by multiplying the average number of cigarettes per day with the number of years smoked. Alcohol intake was indexed by usual ethanol intake from wine, beer, and hard liquor in grams per week. Dietary composition was assessed using a 66‐item food‐frequency questionnaire based on the Willett 61‐item questionnaire.^27^ The summary measures derived included dietary lipid content, as indexed by the keys score^28^, ^29^ daily dietary intake of fiber; monounsaturated, polyunsaturated, and saturated fatty acids; total fat carbohydrate, protein, potassium, calcium, and magnesium; total daily energy intake; and percentages of daily total energy intake from monounsaturated, polyunsaturated, and saturated fatty acids, total fat, carbohydrate, and protein. Physical activity was assessed in work, sports, and leisure domains using a modified Baecke questionnaire.^30^, ^31^ Only the summary scores from the sports and leisure questions were used. The score for sports is a summary of the following: (1) the frequency, duration, and an assigned intensity of the sports reported by participants and (2) 3 additional questions on frequency of sweating, general frequency of playing sports, and a self‐rating of the amount of leisure time physical activity compared with others of the same age.^32^ The score for leisure is a summary of the frequency of watching television (scored inversely), walking, bicycling, and walking/biking to work or shopping.^32^

#### Genotyping Data

The ARIC Study genotype data set contains 609 441 SNPs that are genotyped for 8291 participants. We first selected autosomes from white European participants then applied standard quality control procedures to the selected data set. This involved (1) excluding SNPs with a genotyping rate less than 95%, ones that failed the Hardy–Weinberg test at the 0.0001 level or had a frequency less than 0.01; (2) excluding individuals who were missing 5% of genotype data; and (3) removing related individuals by excluding 1 person at random using a Bernoulli distribution with a selection probability of 0.5, from each pair that had an estimated genomic relationship^33^ greater than 0.05. Eventually, 586 257 SNPs and 7513 individuals remained. Among these individuals, 6896 to 7180 have non missing phenotypic records to be used in the analyses of the 23 traits.

### UK Biobank

The UKBB contains health‐related data from ≈500 000 participants aged between 40 and 69 years who were recruited throughout the United Kingdom between 2006 and 2010.^34^ For validation purposes, we only selected cardiovascular‐related phenotypes and lifestyle covariates that overlapped with the ARIC Study data set, which included BMI, waist‐to‐hip ratio, heart rate, white blood cell count, diastolic and systolic blood pressure, pulse pressure, high‐density lipoprotein (HDL) cholesterol level, apolipoprotein a1 level, smoking (pack years of smoking as proportion of life span exposed to smoking), alcohol intake (average weekly intake of all types), physical activity (metabolic equivalent minutes for walking, moderate activity, vigorous activity, and all types^35^), and dietary composition (polyunsaturated fatty acid, saturated fatty acid, and total energy intake).

#### Genotyping Data

The second release of the UKBB genotyping data set was used. Before quality control, the dataset contains 92 693 895 imputed autosomal SNPs of which genotypes are available for 488 377 individuals. We selected the third phase of the International HapMap project (HapMap3) SNPs from individuals of white British ancestry only and applied the same quality control procedures as for the ARIC Study genotyping data set. Only HapMap3 SNPs were selected because they were shown to yield reliable and robust estimates of SNP‐based heritability and genetic correlation.^36^, ^37^, ^38^ In addition, ambiguous and duplicated SNPs and SNPs with an information score (used to index the quality of genotype imputation) <0.6 were excluded. We computed the genomic relationship matrix of all observations and excluded population outliers, defined as individuals who have a score outside 3 SD on either the first or second principal component of the genomic relationship matrix. From the remaining participants, only those who were part of the first release of the UKBB genotyping data (≈150 000 individuals) were selected for the purpose of reducing computational burden. This subset of participants therefore has 2 versions of imputed genotyping records, 1 from each release, which enabled the computation of discordance rates between the 2 versions for each SNP across individuals and for each individual across SNPs. SNPs and individuals who have a discordance rate >0.05 were excluded. Eventually, 1 130 918 SNPs for 66 281 participants remained. Among these participants, only 14 076 to 34 538 have phenotype data available for analysis.

### Statistical Analysis

#### Interaction Effects Detection

**Figure 1:**
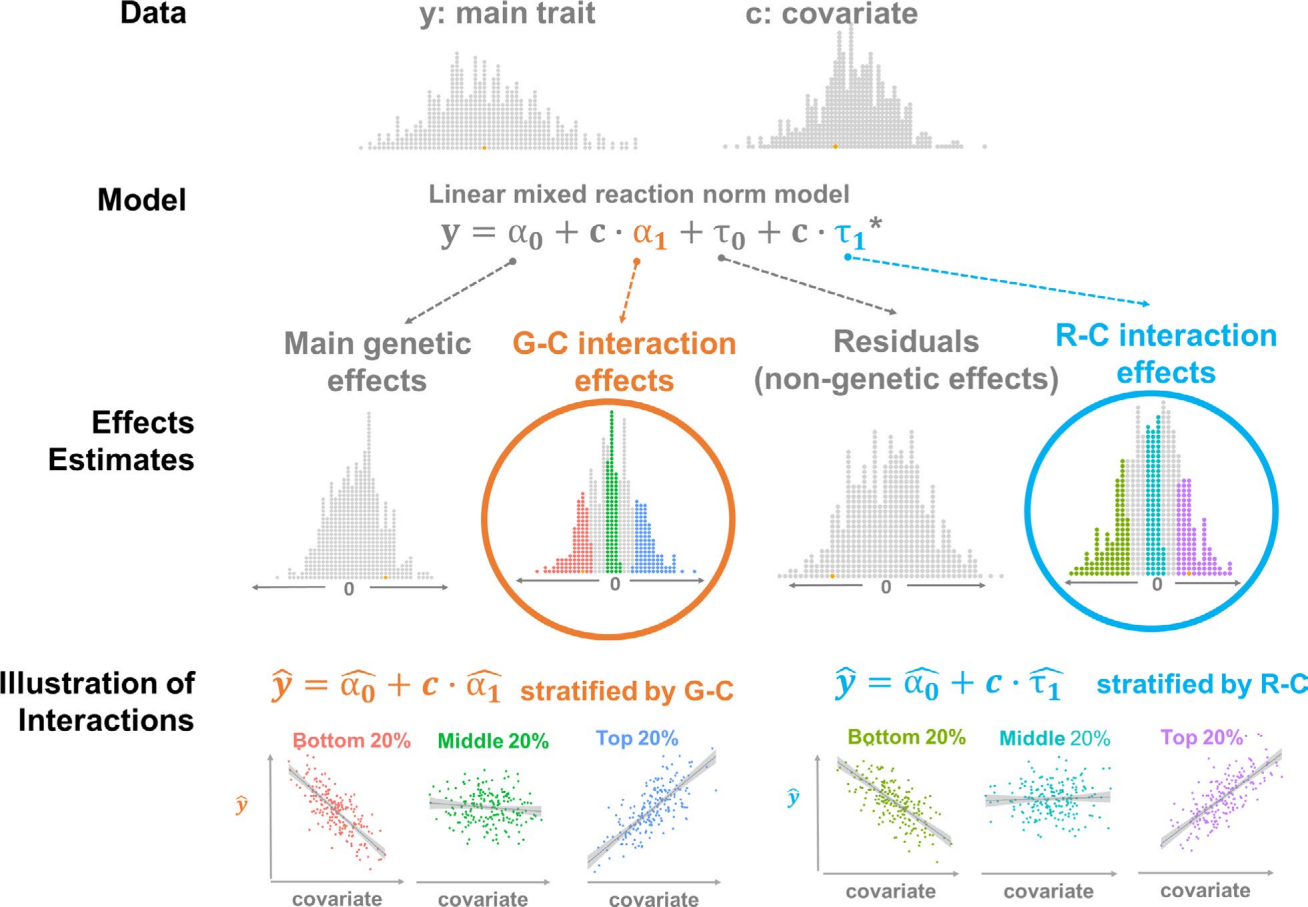
**A schematic illustrating the working of the linear mixed reaction norm model used to study genotype–covariate (G‐C) and residual–covariate (R‐C) interaction effects.** Given phenotypic data of a main trait and a covariate, a reaction norm model that assumes G‐C and R‐C interactions (ie, a full model) decomposes the phenotypic variance of the main trait into genetic variance, residual variance, and their subcomponents that are modulated by the covariate, that is, variance of G‐C interaction effects and variance of R‐C interaction effects. The model then generates per‐individual estimates of main genetic effects, G‐C interaction effects, residuals, and R‐C interaction effects, which can be used to compute estimated phenotypes of the main trait, denoted as $\hat{y}$. When stratified according to interaction effect estimates, $\hat{y}$ and covariate shows different relationships across stratified groups, illustrating G‐C and R‐C interaction effects. For simplicity, only the key part of the linear mixed reaction norm model is shown. *Note that α_1_ and τ_1_ are n×1 vectors of interaction effects of 2 types, that is, G‐C and R‐C interactions, respectively, which serve to capture the heterogeneity of genetic variance and the heterogeneity of residual variance across different values of a given covariate. The 2 effects have different variance–covariance structures. Specifically, $\text{var}\left(\alpha_{1}\right)={\text{WW}}^{T}\sigma_{\alpha 1}^{2}/m$ and $\text{var}\left(\tau_{1}\right)=I\sigma_{\tau 1}^{2}$, where *W* is an *n*×*m* matrix that stores standardized genotypes of *m* single nucleotide polymorphisms for n individuals, noting that WW^T^ is the genomic relationship matrix, and *I* is an *n*×*n* identity matrix.

To estimate the variances of G‐C and R‐C interaction effects, we used multivariate reaction norm models (MRNMs),^10^ where the 2 types of interaction effects are treated as random. Details of MRNMs can be found in Data S1. Briefly, under this approach, the presence of a type of interaction effect is evidenced by its nonzero variance, and we declared nonzero variances of interaction effects when the full model, that is, a MRNM that assumes the presence of G‐C and R‐C interactions, had a better fit than the null model, that is, a MRNM that assumes no G‐C and R‐C interactions. The full model is illustrated graphically in Figure 1, simulation studies used to calibrate our model comparison approach are documented in Data S2 (Figures S1–S4, Tables S1 and S2), and the justification for the model comparison approach is included in Data S3 (Figure S5, Tables S3 and S4). All model comparisons were based on likelihood ratio tests. For our primary analyses, we applied the full model versus the null model comparison approach to the ARIC Study data set. To validate significant results that emerged from the ARIC Study data set, we repeated the analyses using the UKBB for cardiovascular traits where the 2 data sets overlap.

Of note, the full model versus the null model comparison method on its own does not disentangle orthogonal G‐C or R‐C interaction effects. As documented in Data S3 (Figure S5, Tables S3 and S4), we initially considered a series of model comparisons to disentangle orthogonal G‐C or R‐C interaction from overall interactions, but the power of this approach was low (< 11% for interaction effects of a small size; Data S3, Figure S5, Tables S3 and S4). Subsequently, we used the full model comparison versus the null model comparison to detect overall interactions and used parameter estimates from the full model to quantify G‐C and R‐C interactions. This strategy is based on 2 observations from our simulation studies (Data S2, Figures S1–S4, Tables S1 and S2). First, the full model versus the null model comparison in general had a high power of detecting any interaction type or both (>84% even for small interaction effects; Data S2, Figures S1–S4, Tables S1 and S2). Second, the full model consistently yielded unbiased variance component estimates under all simulation scenarios and parameter settings (Data S2, Figures S1–S4, Tables S1 and S2). Importantly, an interaction effect on its own—whether it is a G‐C interaction or an R‐C interaction—has important clinical relevance to cardiovascular risk reduction (as shown later).

#### Phenotype Adjustment

Before fitting MRNMs, all selected cardiovascular traits were adjusted for demographic and lifestyle variables using linear models that regressed phenotypes on demographic and lifestyle variables. The demographic variables included age, sex, education level, marital status, field center identification, and population structure, as measured using the first 15 principal components of the estimated genomic relationship matrix. All lifestyle variables described in the previous section were included for the adjustment. Depending on the cardiovascular trait, some additional adjustment factors were also included. For resting heart rate, blood pressure measures, electrocardiography variables, and coagulation factors, additional adjustment factors included hypertension, defined as systolic blood pressure ≥140 or diastolic blood pressure ≥90, and hypertension‐lowering medication use. For total cholesterol and triglycerides levels, additional adjustment factors were hypertension, hypertension‐lowering medication use, cholesterol‐lowering medication within 2 weeks, and medications that secondarily affect cholesterol. As the second trait in the multivariate reaction normal model (see the second part of Equation 1 in Data S1 where lifestyle covariate is on the left side of the equation), lifestyle covariates were also adjusted in the same way as the cardiovascular trait in the first part of Equation 1 in Data S1.

#### Lifestyle Covariates Versus Nongenetic Determinants of Cardiovascular Traits

Classically, lifestyle covariates have been considered nongenetic determinants of cardiovascular health. In this study, lifestyle covariates and nongenetic determinants are separate concepts and serve as distinct components in our linear mixed reaction norm model (RNM). Specifically, nongenetic effects, in contrast to genetic effects, are referred to as effects of unknown factors that are not explicitly specified in our model and hence are partitioned as residual effects. On the other hand, lifestyle covariates are known factors and hence are explicitly specified in our model as modulators of genetic and nongenetic effects. The distinction is shown in Figure 1.

#### Heritability Estimation

We also considered the consequence of neglecting G‐C and R‐C interactions on heritability estimates. Specifically, we estimated heritability of each trait using 2 models, one that includes no interaction term at all, that is, the null model (that uses genomic restricted maximum likelihood [GREML] for parameter estimation), and the other, referred to as the “interaction model,” that includes significant interaction terms that emerged from our primary analyses, and compared the estimates of the 2 models. To reduce computational burden, we used univariate RNMs as opposed to MRNMs. Details of univariate RNMs can be found in Data S1.

## Results

### G‐C and R‐C Interactions

**Figure 2:**
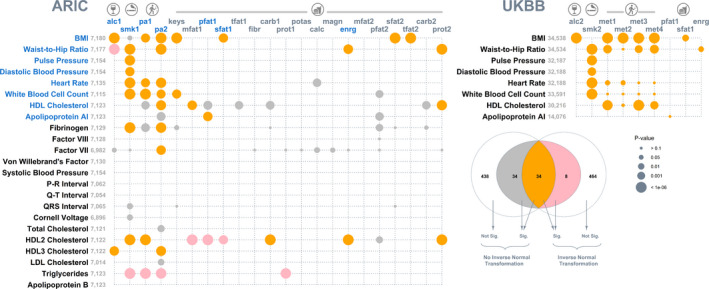
**Bubble plot of *P* values that identify lifestyle modulation of genetic and nongenetic effects on cardiovascular traits.** For each of the 23 cardiovascular traits (along the *y* axis) from the ARIC Study, 22 lifestyle covariates (along the *x* axis) were screened separately for genotype–covariate and residual–covariate interactions by comparing a multivariate reaction norm model that allows genotype–covariate and residual–covariate interactions (ie, a full model) with a null model that assumes no genotype–covariate and residual–covariate interactions (**left**). The 506 null model versus full model comparisons were repeated after a rank‐based inverse normal transformation was applied to all traits for a sensitivity analysis. Signals (after Bonferroni correction) for data before and after the transformation are color coded, as detailed in the Venn diagram (**bottom right**). A total of 34 signals (in orange) remained after the sensitivity analysis. Of these remaining signals, 17 were subject to validation using the UKBB, and their corresponding traits and lifestyle covariates are highlighted in blue. The results of the UK biobank validation are shown (**top right**). For both data sets, bubbles are proportional to *P* values based on data after the rank‐based inverse normal transformation. Note the exceptions to the sample size displayed for BMI versus sfat1 (N=16 257) and for waist‐to‐hip ratio versus enrg (N=16 254) in the UKBB because of the limited availability of dietary intake data among the selected participants. alc1 indicates alcohol intake (g/week); alc2, alcohol intake (glass and pint/week); ARIC, Atherosclerosis Risk in Communities; BMI, body mass index; calc, calcium intake (mg/d); carb1, carbohydrate intake (g/d); carb2, energy from carb1 (%kcal/d); enrg, total energy intake (kcal/d); fibr, dietary fiber intake (g/d); HDL, high‐density lipoprotein; keys, keys score; LDL, low‐density lipoprotein; magn, magnesium intake (mg/d); met1, summed metabolic equivalent minutes/week for all activity; met2, metabolic equivalent minutes/week for walking; met3, metabolic equivalent minutes/week for moderate activity; met4, metabolic equivalent minutes/week for vigorous activity; mfat1, monounsaturated fatty acid intake (g/d); mfat2, energy from mfat1 (%kcal/d); pa1, physical activity: leisure domain; pa2, physical activity: sports domain; pfat1, polyunsaturated fatty acid intake (g/d); pfat2, energy from pfat1 (%kcal/d); potas, potassium intake (mg/d); prot1, protein intake (g/d); prot2, energy from prot1 (%kcal/d); sfat1, saturated fatty acid intake (g/d); sfat2, energy from sfat1 (%kcal/d); Sig., significant; smk1, cigarette years of smoking; smk2, pack years adult smoking as proportion of life span exposed to smoking; tfat1, total fat intake (g/d); and tfat2, energy from tfat1 (%kcal/d); and UKBB, UK Biobank.

We had a total of 23 CVD traits, and for each trait we screened 22 available lifestyle covariates for G‐C and R‐C interactions. Of the 506 pairs of cardiovascular traits and lifestyle covariates, 214 yielded significant results at the 0.05 level, where the full model had a better fit than the null; after Bonferroni correction, 68 pairs remained significant (Figure 2). Of these, 34 survived a sensitivity analysis, where we applied a rank‐based inverse normal transformation to all traits and refit our models. In a further investigation, we noted that a large majority of the signals that were lost after the rank‐based inverse normal transformation were from traits that have large skewness and kurtosis (Figure S6). Given that rank‐based inverse normal transformation can control type I error rate when the normality assumption of MRNM is violated, as shown by the simulation results (Data S2, Figures S1–S4, Tables S1 and S2), the lost signals are likely to be spurious. Hence, in the following we focus on signals that remained after the rank‐based inverse normal transformation.

Of the 34 significant pairs remaining after the rank‐based inverse normal transformation, 17 were covered by the UKBB, allowing replication of the analyses conducted in the ARIC Study. The majority of these signals, 14 of 17, were present in both data sets (Figure 2, right). The 3 signals lost in the replication were the modulating effects of physical activity on white blood cell count and of polyunsaturated fatty acid intake on apolipoprotein a1. In addition, among the replicated signals, the results for physical activity varied slightly when metabolic equivalents were broken down into walking and moderate and vigorous activities, indicating that the modulating effects of physical activity may be conditional on the type of activity. The variance estimates from the full model for all signals from the ARIC Study and UKBB data sets are listed in Tables S5 and S6, respectively. In summary, our results indicate that lifestyle factors that include alcohol intake, smoking, physical activity, and dietary composition are highly relevant to interindividual variability in cardiovascular health and hence CVD risk.

**Figure 3:**
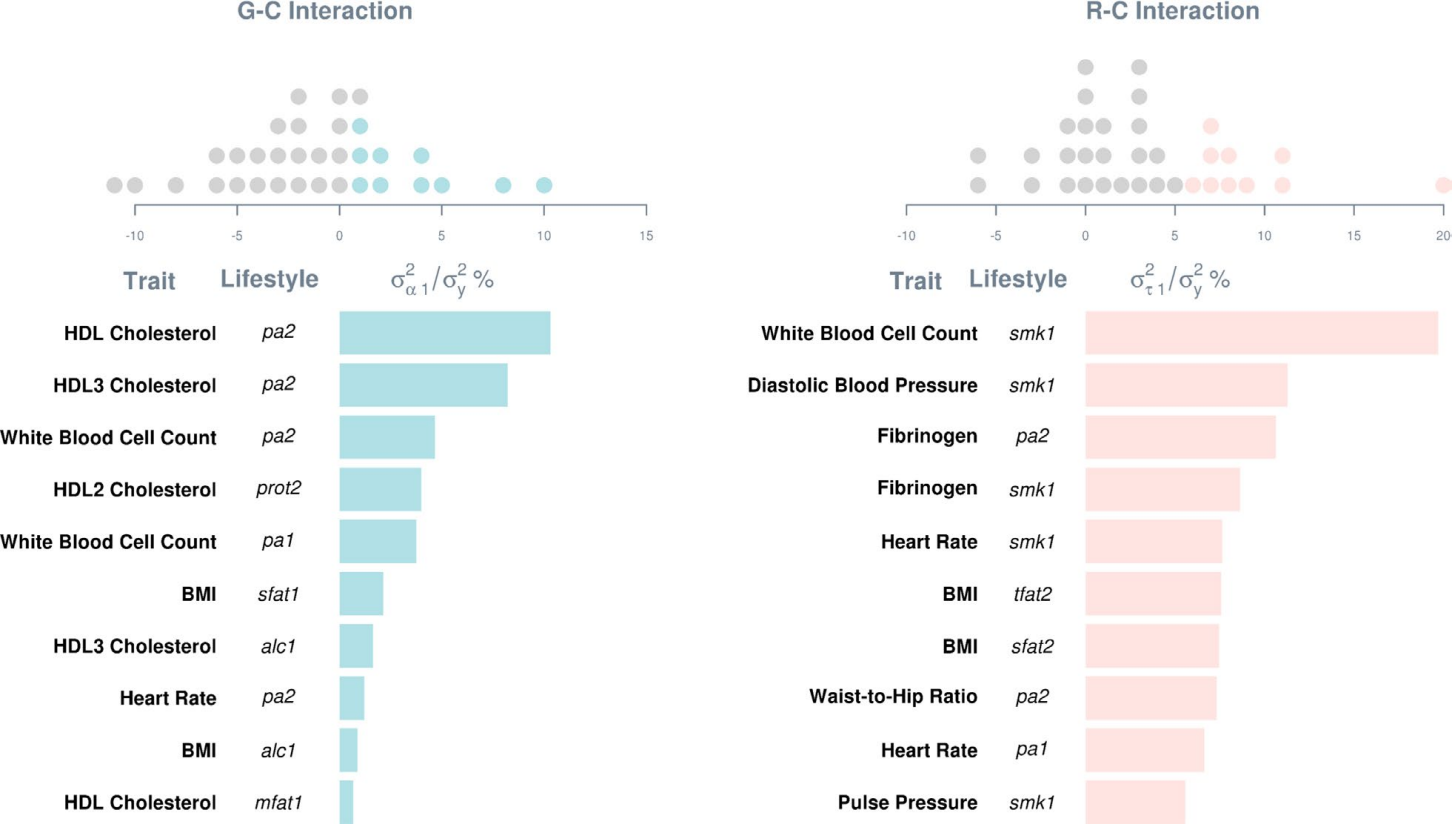
Variance estimates of G‐C and R‐C interactions as percentages of total phenotypic variance.Estimates were derived by fitting a multivariate reaction norm model that included both G‐C and R‐C interactions (ie, a full model) to data without a rank‐based inverse normal transformation. Dot plots on the top show distributions of estimates relative to the phenotypic variance of respective traits. Estimates are included only for signals that remained after a sensitivity analysis, where the full model was better than the null after Bonferroni correction on data after a rank‐based inverse normal transformation. Top ten estimates are shown in bar charts below. *alc1* indicates alcohol intake (g/week); BMI, body mass index; G‐C, genotype–covariate; HDL, high‐density lipoprotein; mfat1, monounsaturated fatty acid intake (g/d); pa1, physical activity: leisure domain; pa2, physical activity: sports domain; prot2, energy from protein intake (%kcal/d); R‐C, residual–covariate; $\sigma_{\alpha 1}^{2}$, variance of G‐C interaction effects; $\sigma_{y}^{2}$, phenotypic variance; $\sigma_{\tau }^{2}$, variance of R‐C interaction effects; sfat1, saturated fatty acid intake (g/d); sfat2, energy from saturated fatty acid intake (%kcal/d); smk1, cigarette years of smoking; and tfat2, energy from total fat intake (%kcal/d).

For the 34 signals emerged from the ARIC Study, magnitudes of G‐C and R‐C interactions were further examined by expressing estimated variances of the 2 types of interactions relative to total phenotypic variances, as shown in Figure 3. Four major observations emerged. First, G‐C and R‐C interactions are sizeable, which can account for up to 20% of phenotypic variance, highlighting the importance of lifestyle modulation to interindividual variability in cardiovascular health. For any given trait, the larger the variance estimate of interaction effects (denoted as $\hat{\sigma_{\alpha 1}^{2}}$ and $\hat{\sigma_{\tau 1}^{2}}$ for G‐C and R‐C interaction effects, respectively), the greater the genetic or residual heterogeneity across different values of the lifestyle covariate, meaning the greater individual differences in the phenotype‐lifestyle relationship; hence stronger interaction effects. Thus, variance estimates of the interaction effects can serve as measures of interaction effect size and hence are indicative of the relative importance of different lifestyle covariates to a given trait. For example, the variance estimate of G‐C interactions of HDL3 cholesterol level is larger for physical activity (abbreviated as pa2) than for alcohol consumption (alc1; see Figure 3, left). Hence, phenotypic changes in HDL3 cholesterol are larger with respective to physical activity than those with respect to alcohol consumption. Similarly, the magnitude of R‐C interactions is larger for fibrinogen–physical activity analysis than for fibrinogen‐smoking analysis (smk1; see Figure 3, right). Standardizing cardiovascular traits also makes across‐traits comparisons meaningful for the same lifestyle covariate. For example, phenotypic changes in white blood cell count are more prone to the modulation of smoking than to the modulation of fibrinogen because the variance estimate of R‐C interactions is larger from the white blood cell count–smoking analysis than from the fibrinogen–smoking analysis.

Second, variance estimates of R‐C interactions are in general larger than G‐C interactions, indicating that lifestyle covariates play a greater role in modulating nongenetic effects on cardiovascular health than genetic effects. Third, some variance estimates can be zero or even below zero. This is not totally unexpected, though, and is within the observed range of sampling errors from analyses of the simulated data (see Data S2, Figures S1–S4, Tables S1 and S2). Lastly, we noted a strong inverse correlation between variance estimates of R‐C and G‐C interactions (Pearson *r*=−0.81). Such collinearity is likely attributed to the same covariate being used for estimating G‐C and R‐C interactions. Similar observations were noted in each replicate of simulated data, yet both variance estimates of G‐C and R‐C interactions were unbiased (see Data S2, Figures S1–S4, Tables S1 and S2). Thus, despite the collinearity between variance estimates, estimation accuracy did not appear to be adversely affected. It is noted that the statistical power to separate G‐C and R‐C interactions can be low, and parameter estimates from models including only G‐C or R‐C interaction (referred to as “G‐C only” and “R‐C only” models) can be biased as shown in simulations (see Data S2, Figures S1–S4, Tables S1 and S2). Consequently, only the null model versus the full model comparison was chosen to indicate lifestyle modulation. Nonetheless, we compared nested models, that is, a G‐C only model and a R‐C only model, with the full model to assess R‐C interaction that is orthogonal to G‐C interaction and G‐C interaction that is orthogonal to R‐C interaction, respectively (Table S7 for the ARIC Study and Table S8 for UKBB).

For the 14 signals that were first discovered in the ARIC Study and replicated in UKBB, we compared variance estimates of G‐C and R‐C interaction effects across the 2 data sets (Tables S5 and S6) and noted some similarities. Physical activity modulates both genetic and nongenetic effects on heart rate and BMI. It also modulates genetic effects on HDL cholesterol level and nongenetic effects on waist‐to‐hip ratio. Alcohol consumption modulates both genetic and nongenetic effects on BMI, whereas smoking modulates nongenetic effects on heart rate, pulse pressure, and white blood cell count. In addition, saturated fat intake modulates genetic effects on BMI, and total daily energy intake modulates nongenetic effects on waist‐to‐hip ratio.

**Figure 4:**
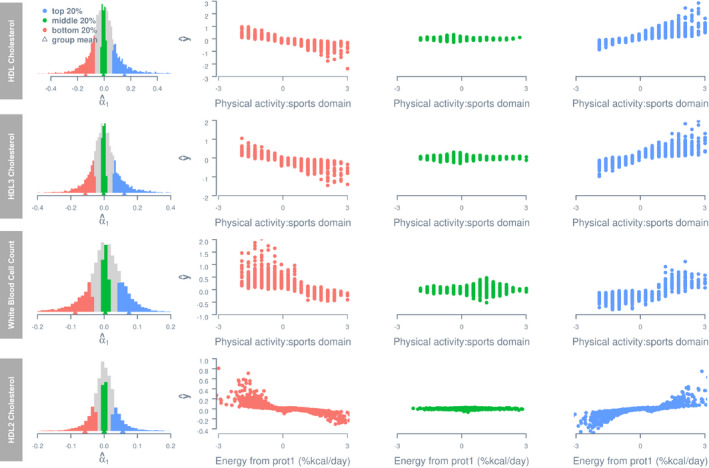
**Estimated phenotypes as a function of lifestyle covariates for groups stratified by per‐individual estimate of genotype–covariate interaction effect.** Histograms on the left show distributions of per‐individual estimates of a genotype–covariate interaction effect, that is, α_1_. The estimated phenotype of any given individual *i* is computed using the equation ${\hat{y}}_{i}={\hat{\alpha }}_{0i}+c_{i}\;{\hat{\alpha }}_{1i}$, where *c* denotes the lifestyle covariate value recorded for *i*, and ${\hat{\alpha }}_{0i}$ and ${\hat{\alpha }}_{1i}$ denote the estimated main genetic effect and genotype–covariate interaction effect for *i*. Only the first 4 traits with the largest variance estimate of genotype–covariate interaction effects are shown. All phenotypes and lifestyle covariates are standardized. HDL, high‐density lipoprotein; and prot1, protein intake (g/d).

The presence of G‐C and R‐C interactions indicates heterogeneity of genetic and residual variance–covariance structures with respect to lifestyle covariates,^39^ which are depicted in Figure S7 for G‐C interactions and in Figure S8 for R‐C interactions. To explicitly illustrate G‐C interactions, for each of the 8 traits with the largest variance estimates of G‐C interaction, we stratified observations into 3 groups—top, middle, and bottom—according to the per‐individual estimate of G‐C interaction effects, denoted as ${\hat{\alpha }}_{1}$ (via the best linear unbiased prediction^40^, ^41^). It is important to note that α_1_ in our model indicates the direction and effect size of the G‐C interaction effect for each individual, and it is assumed to follow a normal distribution with the mean zero. For each trait, we defined the 3 groups as having an ${\hat{\alpha }}_{1}$ below the 20th percentile of the sample (bottom), between the 40th and 60th percentiles (middle), and above the 80th percentile (top), respectively, and plotted their phenotypic estimates, that is, ${\hat{\alpha }}_{0}+\cdot {\hat{\alpha }}_{1}$, given their standardized values on the relevant lifestyle covariate c (Figure 4). Irrespective of the trait, 3 groups showed distinct trajectories of phenotypic changes with respect to lifestyle covariate. Thus, significant G‐C interactions indicated that there exist genetically distinct subpopulations with different phenotype–lifestyle relationships, and hence per‐individual estimates of G‐C interactions inform individual differences, by genetic predisposition, in the extent to which one may benefit from lifestyle changes.

**Figure 5:**
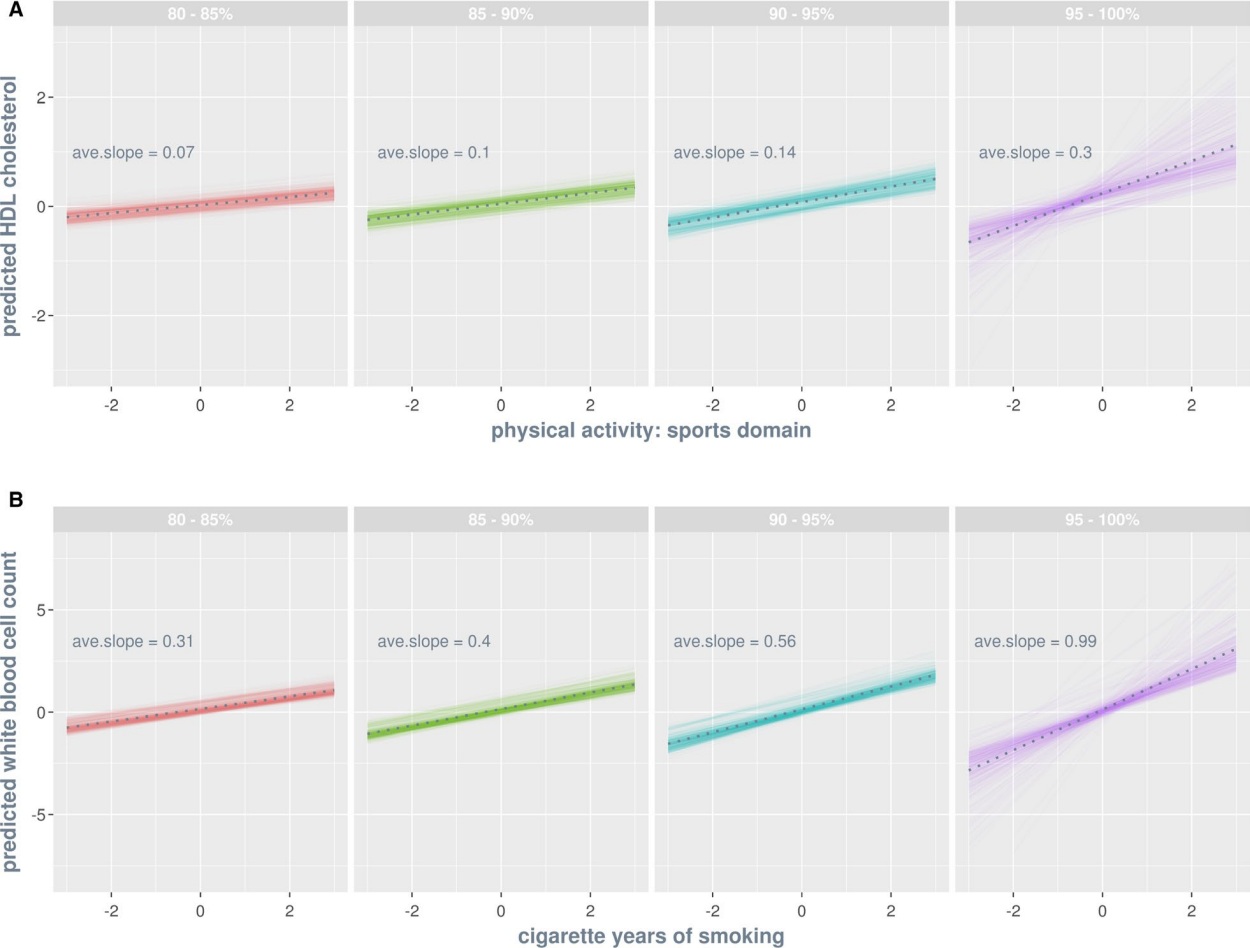
**Predicted trajectories of phenotypic changes as a function of lifestyle covariate by percentile group of estimated genotype–covariate interaction effects (A) and of estimated residual‐covariate interaction effects (B).** Percentile groups are color coded, and within each group faint lines are individuals and the dotted line is the group average. Both traits (shown on the *y* axis) and lifestyle covariates (shown on the *x* axis) are standardized such that the slope of a given trajectory indicates the number of standard unit change in the phenotype of a trait per standard unit change in a lifestyle covariate. **A**, The HDL cholesterol–physical activity analysis is chosen for illustration because it has the largest variance estimate of genotype–covariate interactions. The predicted phenotypes for an individual *i* are computed by substituting lifestyle covariate values, between −3 and 3, into the equation ${\hat{\alpha }}_{0i}+c_{i}{\hat{\alpha }}_{1i}$, where *c* denotes lifestyle covariate, ${\hat{\alpha }}_{0i}$ and ${\hat{\alpha }}_{0i}$ denote the estimated main genetic effect and genotype–covariate interaction effect for *i*, respectively. **B**, The white blood cell count–smoking analysis is chosen for illustration because it is the largest variance estimate of residual‐covariate interactions. The predicted phenotypes for individual *i* are computed by substituting lifestyle covariate values, between −3 and 3, into the equation ${\hat{\alpha }}_{0i}+c_{i}{\hat{\tau }}_{1i}$, where ${\hat{\tau }}_{i}$ denotes the estimated residual‐covariate interaction effect for *i*. ave., average; and HDL, high‐density lipoprotein.

Importantly, G‐C interactions potentially have important clinical relevance. To illustrate, we use the HDL cholesterol–physical activity analysis (with the largest variance estimates of G‐C interaction effects) as an example. Figure 5A shows the predicted trajectories of phenotypic changes in HDL cholesterol level as a function of physical activity for individuals stratified by the percentile group of estimated G‐C interaction (80–85%, 85–90%, 90–95%, and 95–100%). For people with a G‐C interaction estimate that falls between 95% and 100% of the sample, every standard unit increase in physical activity is associated with an average increase in HDL cholesterol level by 0.3 standard unit. This is about 4 times greater than that (0.07 standard unit) for individuals with an interaction estimate that falls between 80% to 85% of the sample. Given the association between HDL cholesterol increase and CVD risk reduction (eg, ref. 42), an increase in physical activity would be most beneficial to individuals in the group that falls between 95% to 100%.

Importantly, the per‐individual estimate of α_1_ that we used for group stratification is an aggregate of G‐C interactions over common SNPs of the whole genome, hence a genome‐wide estimate of G‐C interaction. Therefore, individual differences in ${\hat{\alpha }}_{1}$ would reflect systematic genetic variation, which would be the most pronounced between the 2 extreme groups, that is, top and bottom. A further exploration on pairwise genomic relationships for individuals within and between the 2 extreme groups revealed that the average genomic relationship within each group is greater than the grand average relationship of the entire data set, but the average between‐group relationship is less than the grand average (Table S9). That is, compared with 2 randomly chosen individuals, a pair of within‐group individuals is on average more genetically similar, but a pair of between‐group individuals is on average more genetically distant. This observation holds for all 8 analyses with the largest variance estimates of G‐C interaction (Table S9). Thus, the 2 extreme groups for these analyses in fact have systematic genetic differences.

**Figure 6:**
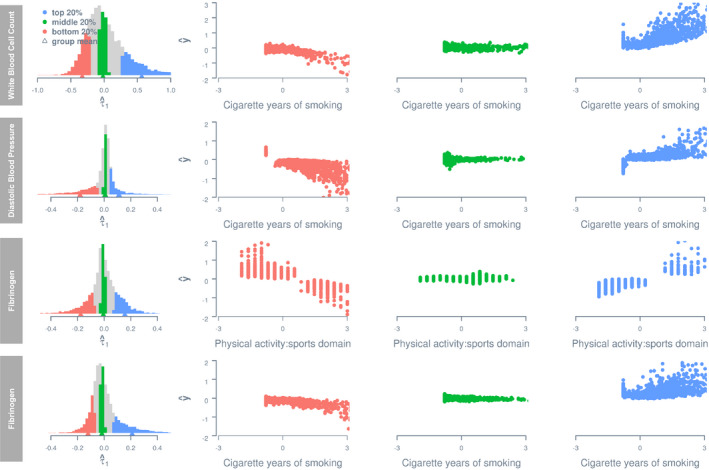
**Estimated phenotypes as a function of lifestyle covariates for groups stratified by per‐individual estimates of residual–covariate interaction.** Histograms on the left show distributions of per‐individual estimates of a residual–covariate interaction effect, τ_1_. The estimated phenotype of any given individual *i* is computed using the equation ${\hat{y}}_{i}={\hat{\alpha }}_{0\;i}+c_{i}{\hat{\tau }}_{1\;i}$, where *c* denotes the lifestyle covariate value recorded for *i*, ${\hat{\alpha }}_{0i}$ and ${\hat{\tau }}_{1i}$ denote the estimated main genetic effect and residual–covariate interaction effect for *i*, respectively. Only the first 4 traits with the largest variance estimate of residual–covariate interaction effects are shown. All phenotypes and lifestyle covariates are standardized.

To explicitly illustrate R‐C interactions, for each of the 8 traits with the largest variance estimates of R‐C interaction, we stratified participants into top, middle, and bottom groups according to per‐individual estimate of R‐C interaction effects, denoted as ${\hat{\tau }}_{1}$, in the same way as for G‐C interaction. Figure 6 shows estimated phenotypes, that is, ${\hat{\alpha }}_{0}+c\cdot {\hat{\tau }}_{1}$, given standardized values on the relevant lifestyle covariate c for the 3 groups. Similar to G‐C interaction, the 3 groups show different phenotypic changes with increasing lifestyle covariate values. Thus, similar to G‐C interactions, R‐C interactions indicate the presence of distinct subpopulations with different phenotype–lifestyle relationships, and hence per‐individual estimates of R‐C interactions inform individual differences in the extent to which one may benefit from lifestyle changes, which are relevant to clinicians and health professionals equally as G‐C interaction estimates.

Figure 5B shows the predicted trajectories of phenotypic changes in white blood cell count as a function of cigarette smoking for individuals stratified by the percentile group of estimated R‐C interaction (80–85%, 85–90%, 90–95%, and 95–100%). For people with a R‐C interaction estimate that falls between 95% and 100% of the sample, every standard unit decrease in smoking is associated with an average reduction in white blood cell count by 0.99 standard unit. This is about 3 times greater than that (0.31 standard unit) for individuals with an interaction estimate that falls between 80% to 85% of the sample. Given the association between white blood cell count decrease and CVD risk reduction (eg, ref. 43), whereas all percentile groups would benefit from a reduction in smoking, the most benefit would be evident for individuals in the 95% to 100% group.

Although a genuine R‐C interaction can be unbiasedly estimated as shown in the simulation (see Data S2, Figures S1–S4, Tables S1 and S2), the models used in this study do not inform how individual differences in ${\hat{\tau }}_{1}$ arise because the fitted variance–covariance structure for residual effects is an identity matrix (see Data S1). However, this problem will no longer exist in a repeated‐measures design or if a nonidentity matrix is fitted for the variance–covariance structure of residual effects.^10^

### Heritability

**Figure 7:**
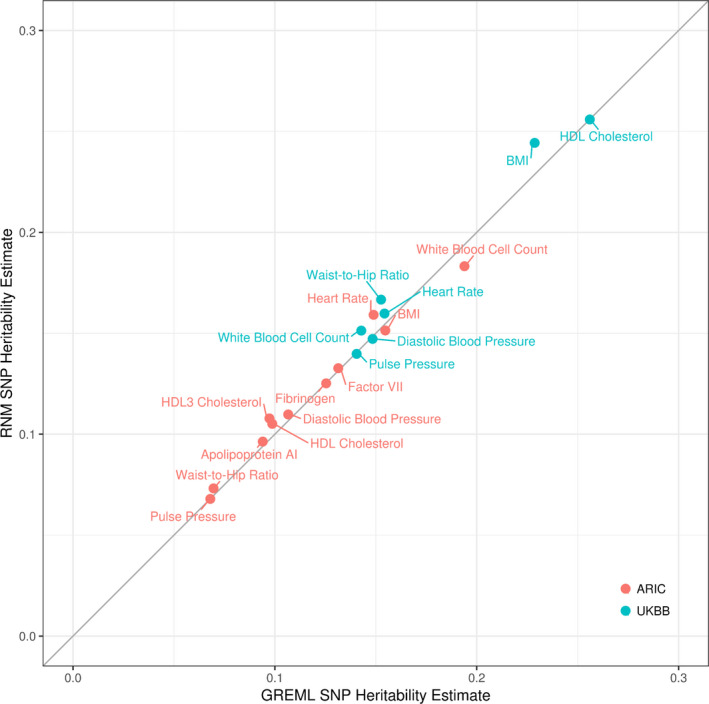
**SNP heritability estimates based on GREML and RNM.** GREML and RNM estimates were derived by fitting a univariate reaction norm model including no interaction term (ie, null model) and one including 1 or more interaction terms (ie, interaction model), respectively. The diagonal is included to highlight the impact of neglecting interaction terms on SNP heritability estimates. Deviations above the diagonal indicate larger RNM estimates relative to GREML estimates. Note HDL2 cholesterol is excluded because of negative variance estimates. ARIC, Atherosclerosis Risk in Communities; BMI, body mass index; GREML, genomic restricted maximum likelihood; HDL, high‐density lipoprotein; RNM, reaction norm models; SNP, single nucleotide polymorphism; and UKBB, UK Biobank.

We showed previously that lifestyle modulation of genetic and nongenetic effects, in forms of G‐C and R‐C interactions, is ubiquitous and sizable for cardiovascular traits. To highlight the importance of incorporating lifestyle modulation when estimating trait heritability, we compared SNP heritability estimates from 2 univariate RNMs (see Methods for details), one without any interaction terms (ie, a null model) and the other with interaction terms (ie, an interaction model) based on results from the MRNMs shown previously (Figure 2). Null model estimates are essentially equivalent to conventional univariate GREML estimates; hence they are referred to as GREML estimates thereafter. As a contrast, interaction model estimates are thereafter referred to as RNM estimates. Figure 7 is a scatter plot of estimates from both the ARIC Study and UKBB data sets. If GREML and RNM estimates are identical, they are expected to align perfectly along the diagonal line. We found that estimates from the interaction model were, on average, slightly yet systematically larger than estimates from the null model (single‐sided paired *t*=2.35, *df*=17, *P*=0.015). Thus, our results support the idea that phenotypic plasticity^39^ can explain some missing heritability (eg, ref. 44).

**Figure 8:**
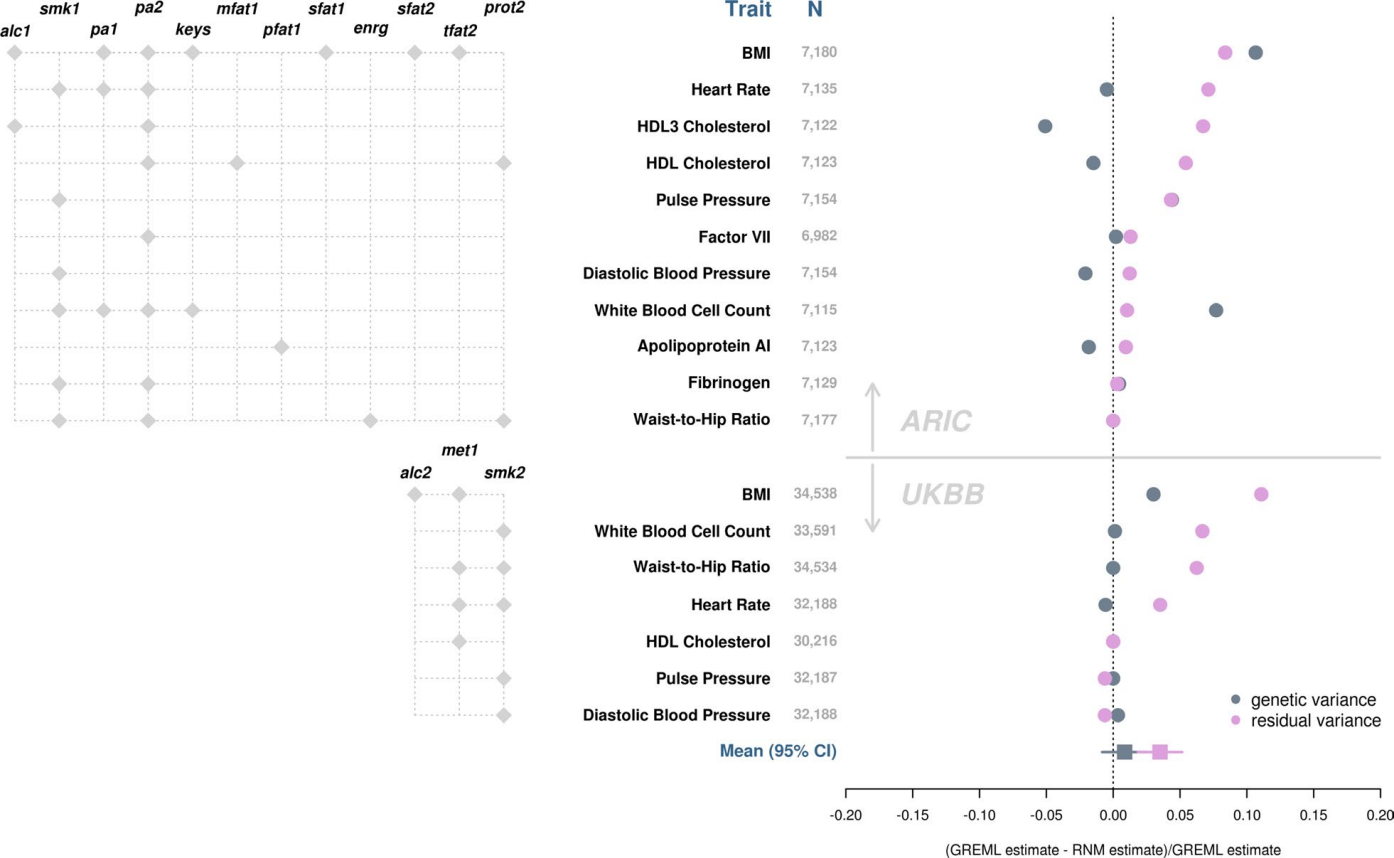
**Genetic and residual variance estimates based on GREML and RNM.** GREML and RNM estimates were derived by fitting a univariate RNM that included no interaction term (ie, null model) and one that included interaction terms (ie, interaction model), respectively. Lifestyle covariate(s) included in the interaction model are specified (**left**). Changes in genetic and residual variance estimates from the interaction model (ie, RNM estimates) relative to their respective estimates from the null model (ie, GREML estimates) are shown to highlight the impact of neglecting interaction terms (**right**). Deviations below 0 indicate underestimation by GREML, whereas deviations above 0 indicate overestimation by GREML. Traits are presented in the decreasing order of deviations for residual variance. Note changes in genetic variance estimates for pulse pressure and waist‐to‐hip ratio in the ARIC Study and for HDL cholesterol in the UKBB are obscured in the plot by data points for residual variance. HDL2 cholesterol in ARIC was excluded because of the negative heritability estimates. alc1 indicates alcohol intake (g/week); alc2, alcohol intake (glass and pint/week); ARIC, Atherosclerosis Risk in Communities; BMI, body mass index; enrg, total energy intake (kcal/d); keys, keys score; GREML, genomic restricted maximum likelihood; HDL, high‐density lipoprotein; met1, summed MET minutes/week for all activity; mfat1, monounsaturated fatty acid intake (g/d); pa1, Physical activity: leisure domain; pa2, physical activity: sports domain; pfat1, polyunsaturated fatty acid intake (kcal/d); prot2, energy from protein intake (%kcal/d); RNM, reaction norm model; sfat1, saturated fatty acid intake (g/d); sfat2, energy from saturated fatty acid intake (%kcal/d); smk1, cigarette years of smoking; smk2, pack years adult smoking as proportion of life span exposed to smoking; tfat2, energy from total fat intake (%kcal/d); and UKBB, UK Biobank.

Given that heritability is a function of genetic and residual variance, we further investigated the reason behind larger RNM heritability estimates by comparing GREML and RNM estimates of genetic and residual variance from both the ARIC Study and UKBB data sets (Figure 8). On average, GREML and RNM estimates of genetic variance were not significantly different, but GREML estimates of residual variance were significantly larger than RNM estimates (see mean and 95% CI in Figure 8; 2‐sided 1‐sample *t*=4.15, *df*=17, *P*=6.7×10^−4^). However, the results from the ARIC Study and UKBB data sets are somewhat different. In particular, some GREML estimates of genetic variance tend to be underestimated for the ARIC Study data, which is not evident for the UKBB data. This is likely attributed to the smaller sample size of the ARIC Study data than the UKBB data, which inevitably results in larger sampling errors for the estimates from the ARIC Study. Nonetheless, our results indicate that G‐C and R‐C interactions are primarily hidden in residual variance estimates in the null model; when they are explicitly estimated in an interaction model, residual variance estimates can be substantially reduced, thereby yielding higher SNP heritability compared with when these components are neglected. This is in line with our previous observation that residual variance is overestimated when fitting a null model to simulated data with genuine G‐C or R‐C interaction.^10^

## Discussion

In this study, we used a novel linear mixed model to detect and estimate components of genetic and nongenetic variance that change with respect to modifiable lifestyle covariates, termed as G‐C and R‐C interactions, in the context of cardiovascular health. Using simulations, we showed that for a sample size of ≈7500 observations, our method has sufficient statistical power to detect genuine G‐C and R‐C interactions while keeping the false positive rate controlled. Applying our method to real data, for each of 23 cardiovascular traits selected from the ARIC Study data set, we screened for G‐C and R‐C interactions using 22 available lifestyle covariates that covered smoking, alcohol intake, physical activity, and dietary composition. G‐C and R‐C interactions were found to be ubiquitous among cardiovascular health related traits, and for some traits, estimates were relatively large, accounting for up to 20% of the total phenotypic variance.

Among the 14 signals replicated in the UKBB, physical activity was found to alter both genetic and nongenetic effects on heart rate and BMI; genetic effects on HDL cholesterol level and nongenetic effects on waist‐to‐hip ratio. Alcohol consumption altered both genetic and nongenetic effects on BMI, whereas smoking altered nongenetic effects on heart rate, pulse pressure, and white blood cell count. In addition, saturated fat intake modified genetic effects on BMI, and total daily energy intake modified nongenetic effects on waist‐to‐hip ratio. To explicitly illustrate G‐C and R‐C interactions, we stratified individuals according to the per‐individual estimate of G‐C and R‐C interactions and showed that genetic and residual effects could take on different directions across groups. Although we did not identify any literature in the context of cardiovascular traits that examined R‐C interaction, the evidence of G‐C interaction in our studies is consistent with the previous literature (eg, refs. 7, 9, 13, 16, 17, 18, 19, 20). Our study is novel in that G‐C interactions were estimated using common SNPs across the entire genome, which are in contrast to estimates based on a single or a limited number of SNPs with large phenotypic effects in past studies.

Given the prevalence of lifestyle modulating effects, we also examined any potential consequence of neglecting these effects on SNP heritability estimates. Such negligence reduced SNP heritability estimates by a small yet significant amount. This reduction is primarily attributed to the overestimation of residual variance. Yet genetic variance estimates are relatively robust to the negligence of significant lifestyle modulation. Our results suggest that current SNP heritability estimates for cardiovascular health‐related outcomes, which commonly do not take into account modulating effects of lifestyle covariates, are likely underestimated.

Currently, several other approaches to G‐C interaction exist in the literature, and our approach is unique in several important ways. Compared with a fixed effects model approach (eg, ref. 45), a mixed‐model approach such as ours could account for genetic covariance among individuals. Compared with StructLMM,^8^ which is a linear mixed‐model approach that examines G‐C interaction for 1 SNP at a time, our approach estimates the G‐C interaction aggregated over SNPs for the entire genome, thereby providing genome‐wide estimates of G‐C interaction. The whole‐genome approach to G‐C interaction also sets this study apart from conventional G‐C interaction studies using a candidate gene approach that focus on only a few genetic variants with large phenotypic effects (eg, refs. 13, 14, 15). In addition, our approach extends other whole‐genome approaches^7^, ^33^, ^46^ by allowing continuous, as opposed to categorical, lifestyle covariates to be modeled and by simultaneously modeling G‐C and R‐C interactions.

The prevalence of the sizable G‐C and R‐C interaction effects shown in our study not only reinforces the relevance of existing lifestyle‐focused prevention programs for CVD prevention but also suggests that promoting lifestyle changes in a single direction may be ineffective or even inappropriate for some subpopulations. Instead, to most effectively reduce genetic and nongenetic predispositions to unfavorable cardiovascular phenotypes, lifestyle‐focused interventions should be tailored to the individual on the basis of his or her relevant genetic and nongenetic information, supporting the rise of precision medicine in CVD to individualize treatments and preventions rather than assuming all individuals share a common pathophenotype.^47^, ^48^

Of note, the variance–covariate structure fitted for the genetic effect in our MRNMs is a nonidentity matrix constructed using genetic information, that is, a genomic relationship matrix. In effect, the SNP best‐linear unbiased predictions derived from MRNMs can be used to predict how a person's genetic risk would change with respect to a chosen lifestyle covariate given his or her genetic information. In contrast, the variance–covariance structure fitted for the residual effect in our MRNMs is an identity matrix. Consequently, R‐C interactions estimated by our models have little use in the prediction of phenotypes. Further development of MRNMs that incorporate a relationship matrix based on factors underlying residual variations, that is, a nonidentity matrix analogous to a genomic relationship matrix, would be useful for the prediction, and it is currently under way in a separate study.

As for other approaches to G‐C interaction for observational studies, the modulating effects of lifestyle covariates found in this study do not imply causality. Although randomized controlled trials are the gold standard, further studies using genetic methods such as Mendelian randomization can help determine causal influences. Furthermore, the MRNMs used in this article are a specific case of the more general MRNMs (see ref. 10), where genetic and residual effects are expanded to the first order of the chosen lifestyle covariate. Higher order expansions may be necessary and could be employed in future studies where the variance–covariance structure for residual effects is a nonidentity matrix. However, increasing model complexity also increases notably the sample size requirement for robust estimation of model parameters. It should also be noted that the sample size in the ARIC Study for our primary analyses is relatively small (6896–7180 participants), leading to less precise parameter estimation compared with the UKBB validation analyses. This may explain some discrepancies observed in the model estimates between the 2 data sets. Smoking, for example, was shown to modulate cardiovascular health in both data sets, but the modulations manifested primarily as R‐C interactions in the ARIC Study analyses but as G‐C interactions in UKBB analyses (see Tables S5 and S6). Independent data sets are required to determine the nature of the modulation effects of smoking on cardiovascular traits. Finally, in this article we only considered intermediate cardiovascular traits that are continuous in nature. The development of valid MRNMs for binary outcome is currently under way. Future applications of these MRNMs would help identify modulating lifestyle covariates that are directly relevant to CVD outcomes.

In summary, we found strong modulations from lifestyle covariates, including smoking, alcohol intake, physical activity, and dietary composition, for genetic and residual effects on phenotypes that are known to associate with CVDs. To illustrate these interactions, we showed that genetic and residuals effects—which may be interpreted as genetic and nongenetic predisposition to CVD health risk, respectively—could change with respect to lifestyle change in different directions for different individuals. Our findings, therefore, reinforce the relevance of lifestyle changes to cardiovascular health and highlight the need for individual considerations when designing lifestyle intervention programs to effectively reduce genetic and nongenetic predispositions to unfavorable cardiovascular phenotypes. Future investigations into specific genetic and nongenetic factors that give rise to individual differences in CVD health risk trajectories with respect to lifestyle changes are well warranted.

## Sources of Funding

This research is supported by the Australian National Health and Medical Research Council (1080157) and the Australian Research Council (DP160102126, DP190100766, FT160100229). The Atherosclerosis Risk in Communities Study has been funded in whole or in part with federal funds from the National Heart, Lung, and Blood Institute, National Institutes of Health, Department of Health and Human Services (under contract numbers HHSN268201700001I, HHSN268201700002I, HHSN268201700003I, HHSN268201700005I, HHSN268201700004I). The UK Biobank is funded by the UK Department of Health, the Medical Research Council, the Scottish Executive, and the Wellcome Trust Medical Research Charity.

## Disclosures

None.

## Supporting information

Data S1–S3
Tables S1–S9
Figures S1–S8

## References

[1] World Health Organization . Fact sheet: cardiovascular diseases. https://www.who.int/en/news-room/fact-sheet​s/detai​l/cardi​ovasc​ular-disea​ses-(cvds). Published May 17, 2017. Accessed February 20, 2020.

[2] Waken RJ , de las Fuentes L , Rao DC . A review of the genetics of hypertension with a focus on gene‐environment interactions. Curr Hypertens Rep. 2017;19:23. 28283927 10.1007/s11906-017-0718-1 PMC5647656

[3] Namboodiri KK , Kaplan EB , Heuch I , Elston RC , Green PP , Rao DC , Laskarzewski P , Glueck CJ , Rifkind BM , Skolnick MH . The collaborative lipid research clinics family study: biological and cultural determinants of familial resemblance for plasma lipids and lipoproteins. Genet Epidemiol. 1985;2:227–254. 4054601 10.1002/gepi.1370020302

[4] Freeman MS , Mansfield MW , Barrett JH , Grant PJ . Genetic contribution to circulating levels of hemostatic factors in healthy families with effects of known genetic polymorphisms on heritability. Arterioscler Thromb Vasc Biol. 2002;22:506–510. 11884298 10.1161/hq0302.104906

[5] Vossen CY , Callas PW , Hasstedt SJ , Long GL , Rosendaal FR , Bovill EG . A genetic basis for the interrelation of coagulation factors. J Thromb Haemost. 2007;5:1930–1935. 17723132 10.1111/j.1538-7836.2007.02678.x

[6] Nowak‐Göttl U , Langer C , Bergs S , Thedieck S , Sträter R , Stoll M . Genetics of hemostasis: differential effects of heritability and household components influencing lipid concentrations and clotting factor levels in 282 pediatric stroke families. Environ Health Perspect. 2008;116:839. 18560491 10.1289/ehp.10754 PMC2430243

[7] Robinson MR , English G , Moser G , Lloyd‐Jones LR , Triplett MA , Zhu Z , Nolte IM , van Vliet‐Ostaptchouk JV , Snieder H , The LifeLines Cohort Study , et al. Genotype–covariate interaction effects and the heritability of adult body mass index. Nat Genet. 2017;49:1174. 28692066 10.1038/ng.3912

[8] Moore R , Casale FP , Jan Bonder M , Horta D , Heijmans BT , C.’t Hoen PA , van Meurs J , Isaacs A , Jansen R , Franke L , et al. A linear mixed‐model approach to study multivariate gene—environment interactions. Nat Genet. 2019;51:180–186. 30478441 10.1038/s41588-018-0271-0 PMC6354905

[9] Young AI , Wauthier F , Donnelly P . Multiple novel gene‐by‐environment interactions modify the effect of FTO variants on body mass index. Nat Commun. 2016;7:12724. 27596730 10.1038/ncomms12724 PMC5025863

[10] Ni G , van der Werf J , Zhou X , Hyppönen E , Wray NR , Lee SH . Genotype–covariate correlation and interaction disentangled by a whole‐genome multivariate reaction norm model. Nat Commun. 2019;10:2239. 31110177 10.1038/s41467-019-10128-w PMC6527612

[11] Maher B . Personal genomes: the case of the missing heritability. Nature. 2008;456:18–21. 18987709 10.1038/456018a

[12] Manolio TA , Collins FS , Cox NJ , Goldstein DB , Hindorff LA , Hunter DJ , McCarthy MI , Ramos EM , Cardon LR , Chakravarti A . Finding the missing heritability of complex diseases. Nature. 2009;461:747. 19812666 10.1038/nature08494 PMC2831613

[13] Hindy G , Wiberg F , Almgren P , Melander O , Orho‐Melander M . Polygenic risk score for coronary heart disease modifies the elevated risk by cigarette smoking for disease incidence. Circ Genom Precis Med. 2018;11:e001856. 29874179 10.1161/CIRCGEN.117.001856 PMC6319562

[14] Khera AV , Emdin CA , Drake I , Natarajan P , Bick AG , Cook NR , Chasman DI , Baber U , Mehran R , Rader DJ , et al. Genetic risk, adherence to a healthy lifestyle, and coronary disease. N Engl J Med. 2016;375:2349–2358. 27959714 10.1056/NEJMoa1605086 PMC5338864

[15] Rutten‐Jacobs LC , Larsson SC , Malik R , Rannikmäe K , MEGASTROKE Consortium, International Stroke Genetics Consortium*,* Sudlow CL , Dichgans M , Markus HS , Traylor M . Genetic risk, incident stroke, and the benefits of adhering to a healthy lifestyle: cohort study of 306 473 UK Biobank participants. BMJ. 2018;363:k4168. 30355576 10.1136/bmj.k4168 PMC6199557

[16] Corella D , Peloso G , Arnett DK , Demissie S , Cupples LA , Tucker K , Lai C‐Q , Parnell LD , Coltell O , Lee Y‐C . Apoa2, dietary fat, and body mass index: replication of a gene‐diet interaction in 3 independent populations. Arch Intern Med. 2009;169:1897–1906. 19901143 10.1001/archinternmed.2009.343 PMC2874956

[17] Latella MC , Di Castelnuovo A , De Lorgeril M , Arnout J , Cappuccio FP , Krogh V , Siani A , Van Dongen M , Donati MB , De Gaetano G . Genetic variation of alcohol dehydrogenase type 1C (adh1c), alcohol consumption, and metabolic cardiovascular risk factors: results from the immidiet study. Atherosclerosis. 2009;207:284–290. 19447389 10.1016/j.atherosclerosis.2009.04.022

[18] Bernstein MS , Costanza MC , James RW , Morris MA , Cambien F , Raoux S , Morabia A . Physical activity may modulate effects of APOE genotype on lipid profile. Arterioscler Thromb Vasc Biol. 2002;22:133–140. 11788473 10.1161/hq0102.101819

[19] Corella D , Guill M , Portol O , Sabater A , Cortina S , Ordovas JM . Environmental factors modulate the effect of the APOE genetic polymorphism on plasma lipid concentrations: ecogenetic studies in a mediterranean Spanish population. Metab, Clin Exp. 2001;50:936–944. 11474482 10.1053/meta.2001.24867

[20] Ruaño G , Seip RL , Windemuth A , Zöllner S , Tsongalis GJ , Ordovas J , Otvos J , Bilbie C , Miles M , Zoeller R . Apolipoprotein A1 genotype affects the change in high density lipoprotein cholesterol subfractions with exercise training. Atherosclerosis. 2006;185:65–69. 16005460 10.1016/j.atherosclerosis.2005.05.029

[21] The ARIC Investigators . The atherosclerosis risk in community (ARIC) study: design and objectwes. Am J Epidemiol. 1989;129:687–702. 2646917

[22] Papp A , Hatzakis H , Bracey A , Wu K . ARIC hemostasis study—I. Development of a blood collection and processing system suitable for multicenter hemostatic studies. Thromb Haemost. 1989;61:015–019. 2526384

[23] Seaman CD , George KM , Ragni M , Folsom AR . Association of Von Willebrand factor deficiency with prevalent cardiovascular disease and asymptomatic carotid atherosclerosis: the atherosclerosis risk in communities study. Thromb Res. 2016;144:236. 27297464 10.1016/j.thromres.2016.05.029 PMC5339890

[24] Folsom AR , Nieto FJ , Sorlie P , Chambless LE , Graham DY . Helicobacter pylori seropositivity and coronary heart disease incidence. Circulation. 1998;98:845–850. 9738638 10.1161/01.cir.98.9.845

[25] O'Neal WT , Singleton MJ , Roberts JD , Tereshchenko LG , Sotoodehnia N , Chen LY , Marcus GM , Soliman EZ . Association between QT‐interval components and sudden cardiac death: the ARIC study (Atherosclerosis Risk in Communities). Circ Arrhythm Electrophysiol. 2017;10:e005485. 29030380 10.1161/CIRCEP.117.005485 PMC5659833

[26] Decker WW , Prina LD , Smars PA , Boggust AJ , Zinsmeister AR , Kopecky SL . Continuous 12‐lead electrocardiographic monitoring in an emergency department chest pain unit: an assessment of potential clinical effect. Ann Emerg Med. 2003;41:342–351. 12605201 10.1067/mem.2003.78

[27] Willett WC , Sampson L , Stampfer MJ , Rosner B , Bain C , Witschi J , Hennekens CH , Speizer FE . Reproducibility and validity of a semiquantitative food frequency questionnaire. Am J Epidemiol. 1985;122:51–65. 4014201 10.1093/oxfordjournals.aje.a114086

[28] Shekelle RB , Shryock AM , Paul O , Lepper M , Stamler J , Liu S , Raynor WJ Jr . Diet, serum cholesterol, and death from coronary heart disease: the western electric study. N Engl J Med. 1981;304:65–70. 7442730 10.1056/NEJM198101083040201

[29] Stamler J , Elliott P , Appel L , Chan Q , Buzzard M , Dennis B , Dyer AR , Elmer P , Greenland P , Jones D . Higher blood pressure in middle‐aged American adults with less education—role of multiple dietary factors: the intermap study. J Hum Hypertens. 2003;17:655. 13679955 10.1038/sj.jhh.1001608 PMC6561108

[30] Baecke JA , Burema J , Frijters JE . A short questionnaire for the measurement of habitual physical activity in epidemiological studies. Am J Clin Nutr. 1982;36:936–942. 7137077 10.1093/ajcn/36.5.936

[31] Folsom AR , Arnett DK , Hutchinson RG , Liao F , Clegg LX , Cooper LS . Physical activity and incidence of coronary heart disease in middle‐aged women and men. Med Sci Sports Exerc. 1997;29:901–909. 9243489 10.1097/00005768-199707000-00009

[32] Bell EJ , Lutsey PL , Windham BG , Folsom AR . Physical activity and cardiovascular disease in African Americans in ARIC. Med Sci Sports Exerc. 2013;45:901. 23247714 10.1249/MSS.0b013e31827d87ec PMC3622814

[33] Yang J , Benyamin B , McEvoy BP , Gordon S , Henders AK , Nyholt DR , Madden PA , Heath AC , Martin NG , Montgomery GW . Common SNPS explain a large proportion of the heritability for human height. Nat Genet. 2010;42:565. 20562875 10.1038/ng.608 PMC3232052

[34] Sudlow C , Gallacher J , Allen N , Beral V , Burton P , Danesh J , Downey P , Elliott P , Green J , Landray M , et al. UK biobank: an open access resource for identifying the causes of a wide range of complex diseases of middle and old age. PLoS Med. 2015;12:e1001779. 25826379 10.1371/journal.pmed.1001779 PMC4380465

[35] Cassidy S , Chau JY , Catt M , Bauman A , Trenell MI . Cross‐sectional study of diet, physical activity, television viewing and sleep duration in 233 110 adults from the UK Biobank; the behavioural phenotype of cardiovascular disease and type 2 diabetes. BMJ Open. 2016;6:e010038. 10.1136/bmjopen-2015-010038 PMC4800116 27008686

[36] Lee SH , Ripke S , Neale BM , Faraone SV , Purcell SM , Perlis RH , Mowry BJ , Thapar A , Goddard ME , Witte JS . Genetic relationship between five psychiatric disorders estimated from genome‐wide SNPs. Nat Genet. 2013;45:984. 23933821 10.1038/ng.2711 PMC3800159

[37] Ripke S , O'Dushlaine C , Chambert K , Moran JL , Kähler AK , Akterin S , Bergen SE , Collins AL , Crowley JJ , Fromer M . Genome‐wide association analysis identifies 13 new risk loci for schizophrenia. Nat Genet. 2013;45:1150. 23974872 10.1038/ng.2742 PMC3827979

[38] Lee SH , Yang J , Chen G‐B , Ripke S , Stahl EA , Hultman CM , Sklar P , Visscher PM , Sullivan PF , Goddard ME . Estimation of SNP heritability from dense genotype data. Am J Hum Genet. 2013;93:1151–1155. 24314550 10.1016/j.ajhg.2013.10.015 PMC3852919

[39] Lynch M , Walsh B . Genetics and Analysis of Quantitative Traits. Sunderland, MA: Sinauer; 1998.

[40] Clark SA , van der Werf J . Genomic best linear unbiased prediction (gBLUP) for the estimation of genomic breeding values. Methods Mol Biol. 2013;1019:321–330. 23756897 10.1007/978-1-62703-447-0_13

[41] Henderson CR . Best linear unbiased estimation and prediction under a selection model. Biometrics. 1975;31:423–447. 1174616

[42] Gordon DJ , Probstfield JL , Garrison RJ , Neaton JD , Castelli WP , Knoke JD , Jacobs DR Jr , Bangdiwala S , Tyroler HA . High‐density lipoprotein cholesterol and cardiovascular disease. Four prospective American studies. Circulation. 1989;79:8–15. 2642759 10.1161/01.cir.79.1.8

[43] Lee CD , Folsom AR , Nieto FJ , Chambless LE , Shahar E , Wolfe DA . White blood cell count and incidence of coronary heart disease and ischemic stroke, and mortality from cardiovascular disease in African‐American and white men and women: the Atherosclerosis Risk in Communities Study. Am J Epidemiol. 2001;154:758–764. 11590089 10.1093/aje/154.8.758

[44] Kaprio J . Twins and the mystery of missing heritability: the contribution of gene‐environment interactions. J Intern Med. 2012;272:440–448. 22934540 10.1111/j.1365-2796.2012.02587.x PMC4422871

[45] Bentley AR , Sung YJ , Brown MR , Winkler TW , Kraja AT , Ntalla I , Schwander K , Chasman DI , Lim E , Deng X , et al. Multi‐ancestry genome‐wide gene‐smoking interaction study of 387,272 individuals identifies new loci associated with serum lipids. Nat Genet. 2019;51:636–648. 30926973 10.1038/s41588-019-0378-y PMC6467258

[46] Dahl A , Nguyen K , Cai N , Gandal MJ , Flint J , Zaitlen N . A robust method uncovers significant context‐specific heritability in diverse complex traits. AJHG. 2020;106:71‐91. 10.1016/j.ajhg.2019.11.015 PMC7042488 31901249

[47] Arena R , Ozemek C , Laddu D , Campbell T , Rouleau CR , Standley R , Bond S , Abril EP , Hills AP , Lavie CJ . Applying precision medicine to healthy living for the prevention and treatment of cardiovascular disease. Curr Probl Cardiol. 2018;43:448–483. 30172550 10.1016/j.cpcardiol.2018.06.001

[48] Leopold JA , Loscalzo J . Emerging role of precision medicine in cardiovascular disease. Circ Res. 2018;122:1302–1315. 29700074 10.1161/CIRCRESAHA.117.310782 PMC6021027

