# Supplementary material for: Whole‐Genome Approach Discovers Novel Genetic and Nongenetic Variance Components Modulated by Lifestyle for Cardiovascular Health
Source: J Am Heart Assoc. 2020 Apr 20;9(8):e015661. doi: 10.1161/JAHA.119.015661 (PMC7428517; doi:10.1161/JAHA.119.015661)
Supplement: Supplementary file 1 — Data S1–S3 Tables S1–S9 Figures S1–S8 [file JAH3-9-e015661-s001.pdf]

Supplemental Material

Data S1. Extended description of statistical methods

Here we provide a description of the statistical models used in the study that is more formal than the description in the main text.

Multivariate Reaction Norm Model

We used a novel whole-genome modelling framework, Multivariate Reaction Norm Models (MRNMs), to detect G-C and R-C interactions. MRNM is an extension of bivariate linear mixed models. In the simplest form of a bivariate linear mixed model, the main trait, *y*, and the covariate, *c*, for individual *i*, after adjusting for their respective fixed effects, μ_y and μ_c, are simultaneously expressed as

$$\begin{pmatrix} y_i - \mu_y \\ c_i - \mu_c \end{pmatrix} = \begin{pmatrix} g_i \\ \beta_i \end{pmatrix} + \begin{pmatrix} e_i \\ \varepsilon_i \end{pmatrix}, \qquad \text{Equation 1}$$

Where g_i ~ N(0, σ_g^2) and β_i ~ N(0, σ_β^2) are genetic effects, which are aggregates of random effects of genome-wide SNPs on the main trait and on the covariate, respectively; e_i ~ N(0, σ_e^2) and ε_i ~ N(0, σ_ε^2) are residual effects, and both g_i and β_i are independent from e_i and ε_i.

MRNM extends Equation 1 by decomposing the random effects of the main traits into main effects and effects modulated by the covariate, which can be written as

$$\begin{pmatrix} y_i - \mu_y \\ c_i - \mu_c \end{pmatrix} = \begin{pmatrix} g_i \\ \beta_i \end{pmatrix} + \begin{pmatrix} e_i \\ \varepsilon_i \end{pmatrix} = \begin{pmatrix} \alpha_{0i} + c_i \cdot \alpha_{1i} \\ \beta_i \end{pmatrix} + \begin{pmatrix} \tau_{0i} + c_i \cdot \tau_{1i} \\ \varepsilon_i \end{pmatrix} \qquad \text{Equation 2}$$

where g_i breaks into $\alpha_{0i} + c_i \cdot \alpha_{1i}$, e_i into $\tau_{0i} + c_i \cdot \tau_{1i}$, α_{0i} ~ N(0, $\sigma_{\alpha 0}^2$), α_{1i} ~ N(0, $\sigma_{\alpha 1}^2$), τ_{0i} ~ N(0, $\sigma_{\tau 0}^2$) and τ_{1i} ~ N(0, $\sigma_{\tau 1}^2$). We use c_i to denote the covariate for individual *i*, α_{0i} for the main genetic effect on the main trait y_i , τ_{0i} for the residual effect, α_{1i} for the genotype-covariate interaction effect, and τ_{1i} for the residual-covariate interaction effect.

As shown in both equations, variance of the main trait and of the covariate are partitioned into two general sources, one of genetics (i.e., σ_g^2 & σ_β^2) and one of non-genetics or residuals (i.e., σ_e^2 & σ_ε^2). By modelling the main trait and the covariate simultaneously, the covariance between the main trait and the covariate, in forms of $cov\ (g_i\ , \beta_i)$ and $cov\ (e_i\ , \varepsilon_i)$, is accounted for in a MRNM. This is important given that the covariance between the main trait and covariate can sometimes be nontrivial and would have been neglected in univariate random regression models. More importantly though, the g_i and e_i terms are expanded in terms of c_i in Equation 2, which offers opportunities to model the genetic and residual variances of the main trait as a function of the covariate. With this expansion, it is immediately clear that genetic variance σ_g^2 breaks into var($\alpha_0 + c \cdot \alpha_1$) and residual variance σ_e^2 into var($\tau_0 + c \cdot \tau_1$), both of which vary with respect to the covariate. As such, MRNMs can estimate and detect genetic and residual variance heterogeneity due to the chosen covariate. A G-C interaction that underlies genetic variance heterogeneity is indicated by significant $\sigma_{\alpha 1}^2$,

and a R-C interaction that underlies residual variance heterogeneity is indicated by significant $\sigma_{\tau 1}^2$.

The MRNM shown in Equation 2 is referred to as the **full** model, which assumes and detects both genetic and residual variance heterogeneity with respect to the covariate. The full model can be simplified into other three major forms. Specifically, by setting both var($c \cdot \alpha_1$) and var($c \cdot \tau_1$) to 0, the **null** model assumes no heterogeneity in either the genetic or the residual variance of the main trait with respect to the covariate. By setting var($c \cdot \tau_1$) to 0, the **G-C** model assumes no R-C interaction and estimates the extent of genetic heterogeneity with respect to the covariate. Finally, by setting var($c \cdot \alpha_1$) to 0, the **R-C** model assumes no G-C interaction and estimates the extent of residual heterogeneity with respect to the covariate.

A detailed description of the variance-covariance structure assumed by MRNMs is provided below. Following Equation 1, the main trait, *y*, and the covariate, *c*, for individual *i*, after adjusting for their respective fixed effects, μ_y and μ_c , are simultaneously expressed as

$$\begin{pmatrix} y_i - \mu_y \\ c_i - \mu_c \end{pmatrix} = \begin{pmatrix} y_i^* \\ c_i^* \end{pmatrix} = \begin{pmatrix} g_i \\ \beta_i \end{pmatrix} + \begin{pmatrix} e_i \\ \varepsilon_i \end{pmatrix}$$

The variance-covariance matrix for *N* realizations of $\begin{pmatrix} y^* \\ c^* \end{pmatrix}$ can be expressed as

$$var\begin{pmatrix} y^* \\ c^* \end{pmatrix} = \begin{bmatrix} \mathbf{Z}_1\mathbf{A}\sigma_{g_1}^2\mathbf{Z}_1' + \mathbf{Z}_1\mathbf{I}\sigma_{e_1}^2\mathbf{Z}_1' & \cdots & \mathbf{Z}_1\mathbf{A}\sigma_{g_{1,N}}\mathbf{Z}_N' + \mathbf{Z}_1\mathbf{I}\sigma_{e_{1,N}}\mathbf{Z}_N' & \mathbf{Z}_1\mathbf{A}\sigma_{g_{1,\beta}}\mathbf{Z}_c' + \mathbf{Z}_1\mathbf{I}\sigma_{e_{1,\varepsilon}}\mathbf{Z}_c' \\ \vdots & \ddots & \vdots & \vdots \\ \mathbf{Z}_N\mathbf{A}\sigma_{g_{1,N}}\mathbf{Z}_1' + \mathbf{Z}_N\mathbf{I}\sigma_{e_{1,N}}\mathbf{Z}_1' & \cdots & \mathbf{Z}_N\mathbf{A}\sigma_{g_N}^2\mathbf{Z}_N' + \mathbf{Z}_N\mathbf{I}\sigma_{e_N}^2\mathbf{Z}_N' & \mathbf{Z}_N\mathbf{A}\sigma_{g_{N,\beta}}\mathbf{Z}_c' + \mathbf{Z}_N\mathbf{I}\sigma_{e_{N,\varepsilon}}\mathbf{Z}_c' \\ \mathbf{Z}_c\mathbf{A}\sigma_{g_{1,\beta}}\mathbf{Z}_1' + \mathbf{Z}_c\mathbf{I}\sigma_{e_{1,\varepsilon}}\mathbf{Z}_1' & \cdots & \mathbf{Z}_c\mathbf{A}\sigma_{g_{N,\beta}}\mathbf{Z}_N' + \mathbf{Z}_c\mathbf{I}\sigma_{e_{N,\varepsilon}}\mathbf{Z}_N' & \mathbf{Z}_c\mathbf{A}\sigma_\beta^2\mathbf{Z}_c' + \mathbf{Z}_c\mathbf{I}\sigma_\varepsilon^2\mathbf{Z}_c' \end{bmatrix}$$

Where $\mathbf{I}$ is an *N* x *N* identity matrix, **A** is the *N* x *N* genomic relationship matrix based on genome-wide SNP information, $\mathbf{Z}_\mathrm{i}$ is the incident matrix for g_i for i = 1, 2 …*N*, and $\mathbf{Z}_\mathrm{c}$ is the incident matrix for $\boldsymbol{c}$.

Prior to model fitting, we attempted to simplify the general MRNMs outlined above by reducing the number of free parameters for estimation. We estimated heritability of each lifestyle covariate in the ARIC dataset via univariate Genomic Restricted Maximum Likelihood (GREML), and found that all estimates were close to zero. Daily potassium intake was the only covariate with an estimate marginally different from zero (h^2 = 0.08 ± 0.04). Subsequently, we simplified MRNMs by setting σ_β^2 (i.e., genetic variance of the covariate) and its associated covariance terms, i.e., $cov\ (\alpha_0\ , \beta)$ and $cov\ (\alpha_1\ , \beta)$, to 0. Unless specified otherwise, all MRNMs fitted to ARIC data in this paper are simplified MRNMs.

For each pair of main trait and covariate, the null and full models were fitted and compared using a likelihood ratio test. For the simplified MRNMs, the test statistic, i.e., -2 log likelihood ratio, is assumed to have a chi-square distribution with five degrees of freedom. The alpha level was set at 0.05. A significant p-value indicates the full

model has a better fit than the null, hence the presence of a G-C, R-C interaction, or both. Since the full model does not separate G-C and R-C interactions, we considered a model comparison strategy to separate the two. However, we show in Data S3 that this strategy can suffer from weak statistical power and biased estimation, which makes it an overall inferior method to the null versus full model comparison method for detecting G-C and R-C interactions. Therefore, our results are based on the latter. All model fitting for this paper was performed using MTG2.

UKBB Validation

To validate significant results found in the ARIC dataset, we repeated analyses using the UKBB for variables where the two datasets overlap. Since the UKBB has a larger sample size, hence greater statistical power, we explicitly estimated the genetic variance of the covariate when fitting a MRNM, rather than fixing this parameter at zero as for the ARIC dataset. Subsequently, the degree of freedom used for the likelihood ratio test that compares the full model with the null model was seven as opposed to five. Same as for the ARIC dataset, we estimated heritability of each UKBB trait using two URNMS (i.e., null and interaction models) and the inclusion of a covariate was based on MRNM results.

Heritability Models

We considered the consequence of neglecting G-C and R-C interactions on heritability estimates. Specifically, we estimated heritability of each trait using two models, one that includes no interaction term at all, i.e., null model (also known as GREML), and the other that includes one or more interaction terms, i.e., interaction model, and compared estimates of the two models. To reduce computational burden, we used univariate reaction norm models (URNMs), as opposed to MRNMs. The null model in the univariate framework is essentially Equation 1 without the part that involves the covariate, c_i. Using the same notation as Equation 1, the main trait for individual i, in a URNM can be written as:

Null model: $$y_i - \mu_y = g_i + e_i$$

The interaction model in the univariate framework expands g_i and e_i as functions of m_1 and m_2 covariates, respectively, where $m_1 + m_2 \geq 1$. Using j to index covariate, the main trait for individual i in a URNM with interaction terms can be written as:

Interaction model: $$y_i - \mu_y = \alpha_{0i} + \sum_{j=1}^{m_1} c_{ij}\alpha_{ij} + \tau_{0i} + \sum_{k=1}^{m_2} c_{ik}\tau_{ik}$$

Data S2. Simulation studies

Simulation Settings

To facilitate data interpretation, we simulated phenotypic data with and without G-C and/or R-C interactions and assessed, using simulated data, whether MRNMs can produce unbiased parameter estimates, type I error rate and power of detecting G-C and R-C interactions. We purposely chose two sets of model parameter configurations that varied primarily in effect size for heritability, G-C and R-C interactions. One setting had large effect sizes, referred to as the 'large-effects setting', with a heritability of 0.5 for both the main trait and the covariate and both $\sigma_{\alpha1}^2$ and $\sigma_{\tau1}^2$, which are indicative of G-C and R-C interactions, were set at 0.5. In contrast, the other setting had smaller effect sizes, referred to as the 'small-effects setting', with a heritability of 0.15 for the main trait, 0 for the covariate, and both $\sigma_{\alpha1}^2$ and $\sigma_{\tau1}^2$ were set at 0.05. It is noted that the small-effects setting resembled more closely parameter estimates from real data analyses than the large-effects setting. Thus, results of the former setting would be more informative about how well our models and the likelihood test for model comparisons perform for analysis of real data.

Each parameter setting covered four scenarios—no G-C and R-C interactions (or the null), R-C interaction only, G-C interaction only, and both R-C and G-C interactions—where the true data generating models were the four models described above. Under each scenario, we simulated 100 replicates of phenotypic data (*n* = 7,513) of a main trait and a covariate, each based on 10,000 randomly chosen causal variants from the ARIC genotype data (see Table S1 for an overview). For every replicate, we fitted the full and null models and compared the fit of the two models using the abovementioned likelihood ratio test. For every scenario, we computed the proportion of replicates, out of 100, for which the full model has a better fit than the null. This proportion takes on different interpretations depending on the simulation scenario. It is an estimate of type I error rate when the true model is the null, whereas it is an estimate of statistical power in scenarios where the true model is other than the null.

It is important to note that all simulating models above assume normally distributed random effects (e.g., genetic and residual effects). In effect, for any given covariate value, the main trait follows a normal distribution. This normality assumption however, is likely violated for many traits of the ARIC and UKBB datasets, which are characterised by substantially larger kurtosis and skewness than would be expected from data simulated under normality (Figure S1). Therefore, in addition to the large and small effects settings described above, we also simulated data with non-normal residuals drawn from Gamma distributions. We purposefully chose two sets of shape and scale parameters of Gamma distributions to represent large and small deviations from normality. For each of the non-normal settings, we had two scenarios: no G-C and R-C interaction (i.e., the null model is true) and G-C and R-C interactions (i.e., the full model is true), each with 100 replicates. We fitted the null and full models, which by definition all assume normality of random effects, to each replicate and subsequently assessed our model comparison method, in terms of type I error, power,

and parameter estimates. In the event of an inflated type I error rate, we applied a rank-based inverse normal transformation to the simulated data and refitted the models. We then assessed the effectiveness of the transformation on reducing false positive findings and its potential consequences on statistical power of the model comparison method and model parameter estimates.

It is important to emphasize that our interaction models do not assume absolute normality of phenotype data, rather their conditional normality on the covariate. Thus, unless the true underlying model is the null, when phenotypic observations are collapsed across covariate values, the distribution of the collapsed data is not necessarily normal. In fact, in the presence of genuine G-C and/or R-C interactions, even when the model normality assumption is met, the simulated phenotype can have larger skewness and kurtosis than data simulated under the null model (see Figure S1). Thus, deviations from normality of a given set of phenotype data could arise from genuine G-C or R-C interaction. If they are mistaken as signs of violation of the model normality assumption, what would be the consequences of applying a rank-based inverse normal transformation for type I error rate, statistical power and model estimates? To answer these questions, we also applied the transformation to phenotype data simulated under normality (i.e., large & small effect parameter settings) and assessed its impact on type I error rate, statistical power and model estimates.

Simulation Results

When the model assumption of normality was met, the estimated type I error rate of the null versus full model comparison method was not inflated (0.04; see Table S2). Small and large phenotypic deviations from the normality inflated the type I error rate to 0.2 and 0.65, respectively. However, after a rank-based inverse normal transformation (RINT) of phenotypic data, the type I error rate was approximately controlled (0.05 and 0.07 for large and small phenotypic deviations from normality, respectively; Table S2), indicating that an RINT can effectively reduce false positive findings in face of violations of the normality assumption held by the MRNMs.

The statistical power of the null versus full model comparison was estimated using data simulated under scenarios other than the null, i.e., G-C only, R-C only and both G-C and R-C interactions. We found that whether the normality assumption is met or not, the proportion of replicates for which the full model had a better fit than the null was at least 0.88 (Table S2), giving an estimated power above 88%. Applying an RINT did not affect the power in any scenario.

For each simulation scenario, we compared parameter estimates from the full model with their corresponding true values. Figure S2 shows sampling distributions of full-model parameter estimates based on 100 replicates for both large and small effects settings (in terms of heritability, G-C and R-C interactions; Table S1) when the model assumption of normality was met, and it indicates that the full model produced unbiased estimates of model parameters under all simulation scenarios. This observation holds even when the normality assumption was violated (Figures S3 & S4). In contrast, after applying an RINT, full-model estimates were biased for some model parameters (Figures S3 & S4).

In summary, our simulation results indicate that when the model assumption of normality is met, the likelihood ratio test that compares the full model with the null can detect G-C and/or R-C interaction at an acceptable type I error rate with a reasonable level of power. When the normality assumption is violated, however, type I error rate would be inflated, in which case an RINT of the phenotype data is an effective remedy without compromising statistical power. In situations where the normality assumption is not violated, a rank-based inverse normal transformation of the phenotype data would not adversely affect type I error rate and statistical power. In terms of parameter estimates, full-model estimates of heritability, G-C and R-C interactions are unbiased, regardless of whether or not the normality assumption is violated. Full-model estimates would however become biased after an RINT. Therefore, for analysis of real data, if the model assumption of normality is in doubt, rank-based inverse transformation should be applied to control type I error rate; and once a significant finding is declared, full model estimates of parameters from data without the transformation should be reported and interpreted.

Data S3. Alternative model comparison strategy

Throughout the text we relied on the null versus full model comparison for detecting G-C and R-C interactions. An alternative and seemingly more logical strategy would be to derive the best model via model comparisons that involve reduced models, i.e., G-C only and R-C only models, in addition to the null and full models. Here we elaborate on this alternative strategy; and using results from simulations, we further show issues associated with this strategy. We conclude that the null versus full model comparison is a superior method, hence a logical choice for detecting G-C and R-C interactions.

This alternative strategy uses result patterns from four model comparisons to conclude the best model out of five candidates (Table S3). Candidates considered were the null model, G-C interaction only model, R-C interaction only model, full model, and G-C or R-C interaction models. The last candidate is more of a situation than a model, where the model comparison method does not distinguish between G-C and R-C models, that is, model selection is inconclusive. It occurs when both G-C model and R-C model show a better fit than the null but a worse fit than the full, that is, G-C and R-C models are equally likely. Upon concluding the best model, the source of variance heterogeneity is immediately implied. For example, genetic variance heterogeneity (i.e., a G-C interaction) is declared, when either the G-C model or the full model is the best. In contrast, residual variance heterogeneity (i.e., a R-C interaction) is declared, when either the R-C model or the full model is the best.

We applied this model selection strategy to simulated data from large- and small-effects settings and evaluated how well the strategy can recover the true simulating model. The results are summarised in Table S4. The method correctly identified the null model for at least 97% of the simulated replicates, giving an estimated type I error rate of 0.03, which is well under the 0.05 target. However, statistical power—estimated by the proportion of replicates for which a true model other than the null is correctly identified—varied largely depending on effect size. For the large-effects setting, a true model other than the null was correctly identified for at least 91% of replicates, hence an estimated power of 0.91 and above. In contrast, for the small-effects setting, the estimated power was 0.04 at worst and 0.11 at best. This does not mean though, the model comparison method could not detect G-C and R-C interactions that are small in magnitude. Rather, for over 75% of replicates under this setting, the likelihood ratio test results were such that G-C and R-C models fit data equally well (see last column of Table S4). In short, either when there are no genuine G-C and R-C interactions or when genuine G-C and R-C interactions are large in magnitude, the likelihood-ratio-based method can discern the true underlying model at a high accuracy (>0.9). However, when genuine G-C and R-C interactions are small, it is unlikely that the method will uncover the true model.

For each simulation scenario, we also compared parameter estimates from all four fitted models with their corresponding true values and noted that results are similar for the two parameter settings that vary in effect sizes. Figure S5 shows results for the small-effects setting, which hold for the large-effects setting. When the true underlying model was the null, regardless of which model was fitted, all parameter estimates were

unbiased. In scenarios where the true model was other than the null, fitting the correct model produced unbiased estimates for all parameters. However, in these scenarios, fitting a wrong model—that is, a model other than the true—could produce biased estimates for some parameters. For example, fitting the null model to data with genuine G-C or/and R-C interactions produced larger estimates of the residual variance, i.e., $\delta_{\tau 0}^{2}$, than its true value, by an amount similar to the set value of $\delta_{\alpha 1}^{2}$ or/and $\delta_{\tau 1}^{2}$. When fitting the G-C model to data with R-C interaction but no G-C interaction, estimates of $\delta_{\alpha 1}^{2}$ were larger than the true, i.e., 0, by an amount similar to the set value of $\delta_{\tau 1}^{2}$. Likewise, when fitting the R-C model to data with G-C interaction but no R-C interaction, estimates of $\delta_{\tau 1}^{2}$ deviated from the true by an amount similar to the set value of $\delta_{\alpha 1}^{2}$. However, fitting the full model, even when it was the wrong model, produced unbiased estimates for all parameters. Thus, our simulation results indicate that model misspecification can result in biased estimates for some parameters depending on the simulation scenario, with the only exception of fitting the full model, which provides unbiased estimates for all parameters in all scenarios.

In summary, the model selection strategy has a type I error rate under 0.05, but its power of recovering the true model is very low when effect sizes are small. Consequently, this strategy can result in an alarmingly elevated chance of concluding a wrong model, i.e., model misspecification, which could produce biased estimates for some model parameters. Therefore, for analysis of real data, where the true underlying model is unknown and effects sizes are likely small, this model comparison strategy is not useful to select the best model for identifying source of variance heterogeneity. In contrast, we showed in the main text that the null versus full model comparison method has an acceptable type I error rate and reasonable power when effect sizes are small. Even if the full model is not true, model estimates are not biased, which can be interpreted subsequently. Hence, the null versus full model comparison is a superior method to the alternative model comparison strategy.

Table S1. True parameter values of four simulation models under four settings.

A. Small effects under Normality

Parameter	*No G-C & R-C*	*G-C & R-C*	*G-C only*	*R-C only*
var(α_0)	0.15	0.15	0.15	0.15
var(α_1)	0	0.05	0.05	0
var(τ_0)	0.85	0.85	0.85	0.85
var(τ_1)	0	0.05	0	0.05
var(β)	0	0	0	0
var(ε)	1	1	1	1
cov(α_0, α_1)	0	0	0	0
cov(τ_0, τ_1)	0	0	0	0
cov(α_0, β)	0	0	0	0
cov(α_1, β)	0	0	0	0
cov(τ_0, ε)	0	0	0	0
cov(τ_1, ε)	0	0	0	0

B. Large effects under Normality

Parameter	*No G-C & R-C*	*G-C & R-C*	*G-C only*	*R-C only*
var(α_0)	0.5	0.5	0.5	0.5
var(α_1)	0	0.5	0.5	0
var(τ_0)	0.5	0.5	0.5	0.5
var(τ_1)	0	0.5	0	0.5
var(β)	0.5	0.5	0.5	0.5
var(ε)	0.5	0.5	0.5	0.5
cov(α_0, α_1)	0	0.05	0.05	0
cov(τ_0, τ_1)	0	0.05	0	0.05
cov(α_0, β)	0	0	0	0
cov(α_1, β)	0	0	0	0
cov(τ_0, ε)	0	0	0	0
cov(τ_1, ε)	0	0	0	0

C. Small Deviation from Normality

Parameter	*No G-C & R-C*	*G-C & R-C*	*G-C only*	*R-C only*
var(α_0)	0.15	0.15	0.15	0.15
var(α_1)	0	0.05	0.05	0
var(τ_0)	0.85	0.85	0.85	0.85
var(τ_1)	0	0.05	0	0.05
var(β)	0	0	0	0
var(ε)	1	1	1	1
cov(α_0, α_1)	0	0	0	0
cov(τ_0, τ_1)	0	0	0	0
cov(α_0, β)	0	0	0	0
cov(α_1, β)	0	0	0	0
cov(τ_0, ε)	0	0	0	0
cov(τ_1, ε)	0	0	0	0
k_0	2	2	2	2
θ_0	0.65	0.65	0.65	0.65
k_1	-	2	-	2
θ_1	-	0.16	-	0.16

D. Large Deviation from Normality

Parameter	*No G-C & R-C*	*G-C & R-C*	*G-C only*	*R-C only*
var(α_0)	0.15	0.15	0.15	0.15
var(α_1)	0	0.05	0.05	0
var(τ_0)	0.85	0.85	0.85	0.85
var(τ_1)	0	0.05	0	0.05
var(β)	0	0	0	0
var(ε)	1	1	1	1
cov(α_0, α_1)	0	0	0	0
cov(τ_0, τ_1)	0	0	0	0
cov(α_0, β)	0	0	0	0
cov(α_1, β)	0	0	0	0
cov(τ_0, ε)	0	0	0	0
cov(τ_1, ε)	0	0	0	0
k_0	0.25	0.25	0.25	0.25
θ_0	1.84	1.84	1.84	1.84
k_1	-	0.25	-	0.25
θ_1	-	0.45	-	0.45

The top two settings are under the assumption that all random effects of the multivariate reaction normal models (MRNMs) for simulation are drawn from normal distributions. This assumption is relaxed for the bottom two settings, where residual effects, τ_0 and τ_1, are drawn from Gamma(k_0, θ_0) and Gamma(k_1, θ_1) with mean centred at zero, respectively. Each setting comprises four simulation models, which from the left to right are the null, full, G-C and R-C models.

Table S2. Proportion of simulated replicates for which the full model had a better fit than the null under different simulation scenarios.

Simulation model	Simulation under Normality				Simulation under Non-Normality				Interpretation
	large effects		small effects		large deviation		small deviation		
	*no RINT**	*RINT*	*no RINT*	*RINT*	*no RINT*	*RINT*	*no RINT*	*RINT*	
No G-C & R-C	0.04	0.04	0.04	0.05	0.65	0.05	0.2	0.07	type I error
G-C only	1	1	0.93	0.94	0.81	1	0.84	0.99	power
R-C only	1	1	0.88	0.87	0.84	1	0.87	0.99	power
G-C & R-C	1	1	1	1	1	1	1	1	power

*RINT = Rank-based Inverse Normal Transformation

Data of a main trait and a covariate were simulated using four models (1st column) under normality with large and small effects (in terms of heritability, Genotype-Covariate (G-C) and Residual-Covariate (R-C) interactions) and under non-normality that resulted in large and small phenotypic deviations of the main trait from normality. Each simulation was repeated 100 times, resulting in 100 replicates of simulated data under each setting. For each replicate, the full model, which allows G-C and R-C interactions, and the null model, which assumes no G-C and R-C interactions, were fitted then compared using a likelihood ratio test. The model comparison was repeated after a rank-based inverse normal transformation was applied to the simulated data.

Table S3. Overview of Model Selection Strategy.

Model Comparison	Candidate Model				
	Null	G-C	R-C	Full	G-C/R-C
Null vs. R-C	×		✓	✓	✓
Null vs. G-C	×	✓		✓	✓
R-C vs. Full		✓	×	✓	×
G-C vs. Full		×	✓	✓	×

Each column shows the model comparison result pattern required to conclude a given candidate model is the best. Result patterns are mutually exclusive across the five candidates. A cross indicates a non-significant p-value for a model comparison (i.e., the simpler model is better), whereas a tick indicates a significant p-value (i.e., the simpler model is worse). Comparisons that are not necessary for model selection are left as blanks. Note the pattern in the last column does not distinguish between Genotype-Covariate (G-C) and Residual-Covariate (R-C) interactions, in which case model selection is inconclusive.

Table S4. Proportions of simulated replicates for which a given candidate model is chosen as the best under different simulation scenarios.

	Simulation Scenario	Candidate Model				
		Null	G-C	R-C	Full	G-C/R-C
large-effects	No G-C & R-C	**0.98**	0	0	0	0.02
	G-C only	0	**0.93**	0	0.07	0
	R-C only	0	0	**0.96**	0.04	0
	G-C & R-C	0	0.03	0.06	**0.91**	0
small-effects	No G-C & R-C	**0.97**	0.01	0.02	0	0
	G-C only	0.06	**0.11**	0.03	0.04	0.76
	R-C only	0.1	0.01	**0.09**	0.05	0.75
	G-C & R-C	0	0.05	0.14	**0.04**	0.77

Data were simulated under normality with large and small effects in terms of heritability, Genotype-Covariate (G-C) and Residual-Covariate (R-C) interactions. Each scenario had 100 replicates of a main trait and a covariate. For each replicate, four models were fitted and compared to select the best fitting one (see Table S3).

Table S5. Variance and covariance estimates from the full model for the 34 signals emerged from the atherosclerosis risk in communities study.

Main Trait	Lifestlye Covariate	var(α_0)	var(α_1)	cov(α_0,α_1)	var(τ_0)	var(τ_1)	cov(τ_0,τ_1)
Fibrinogen	Cigarette years of smoking	4.0e+02(1.5e+02)	-3.6e+02(1.5e+02)	-7.9e+01(1.1e+02)	3.1e+03(1.6e+02)	2.9e+02(1.6e+02)	3.6e+02(1.2e+02)
Fibrinogen	Physical activity:sports domain	4.2e+02(1.5e+02)	-2.5e+02(1.3e+02)	1.5e+01(1.0e+02)	2.9e+03(1.6e+02)	3.6e+02(1.4e+02)	-2.7e+02(1.1e+02)
Factor VII	Physical activity:sports domain	1.0e+02(3.6e+01)	2.6e+00(3.1e+01)	-1.5e+01(2.4e+01)	6.5e+02(3.8e+01)	7.2e+00(3.2e+01)	-4.9e+01(2.5e+01)
BMI	Alcohol intake (g/week)	2.9e+00(9.0e-01)	1.8e-01(6.9e-01)	-1.1e+00(5.7e-01)	1.7e+01(9.3e-01)	5.7e-01(6.9e-01)	-1.3e+00(6.0e-01)
BMI	Physical activity:leisure domain	3.2e+00(9.2e-01)	-6.7e-02(8.3e-01)	3.1e-01(6.3e-01)	1.7e+01(9.6e-01)	5.9e-01(8.7e-01)	-1.3e+00(6.4e-01)
BMI	Physical activity:sports domain	3.1e+00(9.1e-01)	1.1e-01(6.6e-01)	2.3e-02(5.8e-01)	1.7e+01(9.6e-01)	4.2e-01(7.1e-01)	-2.5e+00(6.1e-01)
BMI	Keys score	3.2e+00(9.2e-01)	-4.0e-01(8.0e-01)	-3.3e-01(6.2e-01)	1.7e+01(9.6e-01)	5.5e-01(8.3e-01)	1.5e+00(6.3e-01)
BMI	Saturated fat intake (g/day)	3.1e+00(9.2e-01)	4.3e-01(8.7e-01)	-2.5e-01(6.5e-01)	1.7e+01(9.6e-01)	-5.8e-01(9.1e-01)	1.2e+00(6.6e-01)
BMI	Energy from saturated fat (%kcal/day)	3.1e+00(9.2e-01)	-1.2e+00(8.1e-01)	-5.2e-01(6.2e-01)	1.7e+01(9.6e-01)	1.5e+00(8.5e-01)	1.6e+00(6.4e-01)
BMI	Energy from total fat intake (%kcal/day)	3.1e+00(9.2e-01)	-1.2e+00(8.2e-01)	-3.4e-01(6.3e-01)	1.7e+01(9.6e-01)	1.5e+00(8.8e-01)	1.3e+00(6.4e-01)
Waist-to-Hip Ratio	Cigarette years of smoking	3.0e-04(2.0e-04)	0.0e+00(2.0e-04)	-1.0e-04(1.0e-04)	3.7e-03(2.0e-04)	0.0e+00(2.0e-04)	-1.0e-04(1.0e-04)
Waist-to-Hip Ratio	Physical activity:sports domain	3.0e-04(2.0e-04)	-2.0e-04(1.0e-04)	0.0e+00(1.0e-04)	3.8e-03(2.0e-04)	3.0e-04(2.0e-04)	-2.0e-04(1.0e-04)
Waist-to-Hip Ratio	Total energy intake (kcal/day)	3.0e-04(2.0e-04)	-1.0e-04(1.0e-04)	1.0e-04(1.0e-04)	3.9e-03(2.0e-04)	0.0e+00(2.0e-04)	-3.0e-04(1.0e-04)
Waist-to-Hip Ratio	Energy from protein intake (%kcal/day)	3.0e-04(2.0e-04)	-1.0e-04(2.0e-04)	-1.0e-04(1.0e-04)	3.8e-03(2.0e-04)	0.0e+00(2.0e-04)	2.0e-04(1.0e-04)
Pulse Pressure	Cigarette years of smoking	8.0e+00(5.1e+00)	-1.1e+00(5.5e+00)	-2.7e+00(3.8e+00)	1.0e+02(5.5e+00)	6.3e+00(5.8e+00)	4.6e+00(4.1e+00)
Diastolic Blood Pressure	Cigarette years of smoking	7.4e+00(3.2e+00)	-7.3e+00(3.0e+00)	-2.5e+00(2.2e+00)	6.4e+01(3.4e+00)	8.1e+00(3.2e+00)	4.4e+00(2.4e+00)
Heart Rate	Cigarette years of smoking	1.3e+01(4.1e+00)	-2.1e+00(4.3e+00)	1.9e+00(3.0e+00)	7.1e+01(4.3e+00)	6.8e+00(4.6e+00)	-1.6e+00(3.1e+00)
Heart Rate	Physical activity:leisure domain	1.3e+01(4.1e+00)	-4.4e+00(3.4e+00)	4.9e+00(2.7e+00)	7.4e+01(4.3e+00)	5.9e+00(3.7e+00)	-8.8e+00(2.8e+00)
Heart Rate	Physical activity:sports domain	1.2e+01(4.0e+00)	1.1e+00(3.2e+00)	3.4e+00(2.6e+00)	7.7e+01(4.3e+00)	-4.6e-01(3.4e+00)	-6.7e+00(2.7e+00)
HDL2 Cholesterol	Cigarette years of smoking	-1.0e-04(1.8e-03)	-3.0e-04(1.3e-03)	5.0e-04(1.1e-03)	4.0e-02(1.9e-03)	4.0e-04(1.4e-03)	-3.1e-03(1.1e-03)
HDL2 Cholesterol	Physical activity:leisure domain	-6.0e-04(1.8e-03)	-1.2e-03(1.7e-03)	2.0e-04(1.2e-03)	4.0e-02(1.9e-03)	1.7e-03(1.8e-03)	3.0e-03(1.3e-03)
HDL2 Cholesterol	Carbohydrate intake (g/day)	-5.0e-04(1.8e-03)	-1.1e-03(1.4e-03)	-4.0e-04(1.1e-03)	4.0e-02(1.9e-03)	1.2e-03(1.4e-03)	-1.9e-03(1.1e-03)
HDL2 Cholesterol	Total energy intake (kcal/day)	-4.0e-04(1.8e-03)	-1.5e-03(1.3e-03)	-6.0e-04(1.1e-03)	4.1e-02(1.9e-03)	6.0e-04(1.4e-03)	-9.0e-04(1.2e-03)
HDL2 Cholesterol	Energy from protein intake (%kcal/day)	-4.0e-04(1.8e-03)	1.6e-03(1.6e-03)	-4.0e-04(1.2e-03)	4.1e-02(1.9e-03)	-2.2e-03(1.6e-03)	2.4e-03(1.3e-03)
HDL3 Cholesterol	Alcohol intake (g/week)	5.9e-03(2.7e-03)	1.0e-03(3.3e-03)	-3.9e-03(2.1e-03)	5.5e-02(2.9e-03)	-7.0e-04(3.3e-03)	5.9e-03(2.3e-03)
HDL3 Cholesterol	Physical activity:sports domain	6.2e-03(2.7e-03)	5.0e-03(2.8e-03)	1.4e-03(2.0e-03)	5.1e-02(2.9e-03)	-1.8e-03(2.9e-03)	-2.4e-03(2.0e-03)
HDL Cholesterol	Physical activity:sports domain	1.4e-02(6.1e-03)	1.4e-02(6.3e-03)	4.8e-03(4.3e-03)	1.2e-01(6.6e-03)	-7.8e-03(6.4e-03)	-7.0e-03(4.4e-03)
HDL Cholesterol	Monounsaturated fatty acid intake (g/day)	1.3e-02(6.2e-03)	9.0e-04(5.2e-03)	-2.0e-03(4.0e-03)	1.2e-01(6.5e-03)	-9.0e-04(5.2e-03)	-4.4e-03(4.2e-03)
HDL Cholesterol	Energy from protein intake (%kcal/day)	1.3e-02(6.2e-03)	-4.7e-03(5.9e-03)	1.0e-03(4.4e-03)	1.2e-01(6.6e-03)	5.8e-03(6.1e-03)	4.3e-03(4.5e-03)
Apolipoprotein AI	Polyunsaturated fatty acid intake (g/day)	7.3e+03(3.4e+03)	-3.4e+03(2.9e+03)	3.7e+03(2.1e+03)	6.8e+04(3.5e+03)	3.8e+03(2.9e+03)	-7.4e+03(2.3e+03)
White Blood Cell Count	Cigarette years of smoking	4.1e-01(1.2e-01)	-6.6e-01(4.6e-02)	-3.0e-01(8.0e-02)	3.0e+00(1.4e-01)	5.8e-01(7.4e-02)	8.6e-01(9.2e-02)
White Blood Cell Count	Physical activity:leisure domain	5.7e-01(1.3e-01)	1.1e-01(1.3e-01)	-7.1e-02(9.4e-02)	2.3e+00(1.4e-01)	1.2e-02(1.3e-01)	-1.7e-01(9.5e-02)
White Blood Cell Count	Physical activity:sports domain	5.5e-01(1.3e-01)	1.4e-01(1.3e-01)	-1.3e-01(9.5e-02)	2.2e+00(1.4e-01)	9.1e-02(1.4e-01)	-9.1e-02(9.7e-02)
White Blood Cell Count	Keys score	5.5e-01(1.3e-01)	3.9e-03(1.2e-01)	1.5e-01(9.3e-02)	2.4e+00(1.4e-01)	-1.0e-02(1.3e-01)	3.0e-03(9.3e-02)

All estimates are derived from analyses of data without a rank-based inverse normal transformation. Standard errors are in brackets. Other model parameters are omitted for simplicity. Signals replicated in the UK biobank are shaded. Note the UK biobank only has data available to validate 17 signals emerged from ARIC.

Table S6. Variance and covariance estimates from the full model for the UK Biobank dataset.

Main Trait	Lifestlye Covariate	var(α_0)	var(α_1)	cov(α_0,α_1)	var(τ_0)	var(τ_1)	cov(τ_0,τ_1)
BMI	Alcohol intake (glass & pint/week)	3.9e+00(2.0e-01)	4.4e-01(2.1e-01)	-1.4e-01(1.4e-01)	1.3e+01(2.1e-01)	3.2e-01(2.3e-01)	-4.4e-01(1.6e-01)
BMI	MET minutes/week for walking	4.0e+00(2.0e-01)	5.7e-02(1.7e-01)	-3.6e-01(1.3e-01)	1.2e+01(2.3e-01)	1.1e+00(1.9e-01)	-1.2e+00(1.5e-01)
BMI	MET minutes/week for moderate activity	3.9e+00(2.0e-01)	-1.8e-01(1.4e-01)	-1.9e-01(1.2e-01)	1.2e+01(2.2e-01)	1.4e+00(1.8e-01)	-1.7e+00(1.5e-01)
BMI	MET minutes/week for vigorous activity	4.0e+00(2.1e-01)	1.6e-01(5.7e-01)	-1.6e-01(5.7e-01)	1.3e+01(2.2e-01)	5.8e-01(5.8e-01)	-1.8e+00(5.7e-01)
BMI	Summed MET minutes/week for all activity	3.9e+00(3.7e-01)	-1.1e-01(1.9e-01)	-2.7e-01(2.4e-01)	1.3e+01(2.1e-01)	1.2e+00(1.9e-01)	-1.7e+00(2.2e-01)
BMI	estimated saturated fat intake	3.9e+00(3.7e-01)	4.7e-01(3.6e-01)	-9.6e-02(2.6e-01)	1.3e+01(3.8e-01)	-2.1e-01(3.7e-01)	1.9e-01(2.6e-01)
Diastolic Blood Pressure[1]	Pack years adult smoking as proportion of life span exposed to smoking	1.6e+01(1.2e+00)	2.2e+00(9.5e-01)	-1.8e+00(7.8e-01)	9.1e+01(1.3e+00)	-2.7e+00(9.0e-01)	3.2e+00(8.5e-01)
Pulse Pressure[1]	Pack years adult smoking as proportion of life span exposed to smoking	2.5e+01(2.0e+00)	-9.5e-01(1.8e+00)	2.1e+00(1.3e+00)	1.5e+02(2.2e+00)	2.4e-02(1.7e+00)	2.0e+00(1.5e+00)
Heart Rate	Pack years adult smoking as proportion of life span exposed to smoking	2.0e+01(1.5e+00)	6.0e-01(1.5e+00)	-6.7e-01(1.1e+00)	1.1e+02(1.8e+00)	2.1e+00(1.7e+00)	3.3e+00(1.2e+00)
Heart Rate	MET minutes/week for walking	2.0e+01(1.5e+00)	7.5e-01(1.3e+00)	2.0e-01(9.7e-01)	1.1e+02(1.8e+00)	1.5e+00(1.5e+00)	-3.8e+00(1.2e+00)
Heart Rate	Summed MET minutes/week for all activity	2.0e+01(1.5e+00)	-6.8e-01(1.3e+00)	-6.6e-02(1.0e+00)	1.1e+02(1.7e+00)	4.7e+00(1.5e+00)	-4.5e+00(1.2e+00)
Waist-to-Hip Ratio[2]	Pack years adult smoking as proportion of life span exposed to smoking	1.5e-01(1.1e-02)	-3.6e-03(1.0e-02)	-4.7e-03(7.7e-03)	8.5e-01(1.3e-02)	4.1e-02(1.2e-02)	3.4e-03(9.1e-03)
Waist-to-Hip Ratio	MET minutes/week for moderate activity	1.5e-01(1.1e-02)	5.6e-03(1.0e-02)	-1.2e-02(7.3e-03)	8.1e-01(1.3e-02)	2.9e-02(1.2e-02)	-2.3e-02(9.6e-03)
Waist-to-Hip Ratio	MET minutes/week for vigorous activity	1.5e-01(1.1e-02)	3.6e-03(1.0e-02)	-1.3e-02(7.5e-03)	8.3e-01(1.2e-02)	1.8e-02(1.1e-02)	-2.5e-02(9.4e-03)
Waist-to-Hip Ratio	Summed MET minutes/week for all activity	1.5e-01(1.1e-02)	9.5e-03(1.0e-02)	-1.1e-02(7.5e-03)	8.2e-01(1.3e-02)	1.9e-02(1.1e-02)	-1.9e-02(8.9e-03)
Waist-to-Hip Ratio	estimated total energy intake	1.8e-01(2.2e-02)	-2.0e-02(2.0e-02)	5.0e-03(1.5e-02)	8.1e-01(2.3e-02)	3.3e-02(2.1e-02)	-1.6e-02(1.5e-02)
White Blood Cell Count	Pack years adult smoking as proportion of life span exposed to smoking	5.2e-01(4.2e-02)	-2.7e-02(4.2e-02)	-2.6e-02(3.0e-02)	3.0e+00(5.2e-02)	4.1e-01(5.4e-02)	-1.4e-01(3.6e-02)
HDL Cholesterol	MET minutes/week for moderate activity	2.8e-02(1.4e-03)	1.8e-03(1.3e-03)	5.0e-04(1.0e-03)	8.2e-02(1.6e-03)	-2.4e-03(1.4e-03)	2.4e-03(1.2e-03)
HDL Cholesterol	MET minutes/week for vigorous activity	2.8e-02(1.4e-03)	2.0e-04(1.2e-03)	1.0e-03(1.0e-03)	8.1e-02(1.5e-03)	7.0e-04(1.3e-03)	-1.2e-03(1.1e-03)
HDL Cholesterol	Summed MET minutes/week for all activity	2.8e-02(1.4e-03)	6.0e-04(1.2e-03)	5.0e-04(1.0e-03)	8.2e-02(1.5e-03)	-5.0e-04(1.3e-03)	1.6e-03(1.1e-03)

1.Estimation for the multivariate analysis did not converge. Shown are estimates from an univariate analysis, where the full model had a better fit than the null.

2.Estimates are based on standardized data for this trait, due to small phenotypic variance, which resulted in rather small estimates of variance components in absolute terms.

All estimates are derived from analyses of data without a rank-based inverse normal transformation. Standard errors are in brackets. Other model parameters are omitted for simplicity.

Table S7. P-values for comparisons between the full model and nested models for the atherosclerosis risk in communities study.

Main Trait	Lifestlye Covariate	Null vs. Full	GC vs. Full	RC vs. Full
Fibrinogen	Cigarette years of smoking	4.22E-10	1.83E-03	3.53E-01
Fibrinogen	Physical activity:sports domain	3.27E-06	2.57E-03	2.52E-01
Factor VII	Physical activity:sports domain	2.99E-05	7.19E-01	8.62E-01
BMI	Alcohol intake (g/week)	2.60E-12	5.80E-02	1.83E-01
BMI	Physical activity:leisure domain	9.04E-05	3.29E-01	8.69E-01
BMI	Physical activity:sports domain	9.23E-39	3.30E-08	9.60E-01
BMI	Keys score	1.28E-10	1.45E-01	9.73E-01
BMI	Saturated fat intake (g/day)	5.44E-05	4.66E-01	9.75E-01
BMI	Energy from saturated fat (%kcal/day)	6.24E-10	5.85E-02	3.72E-01
BMI	Energy from total fat intake (%kcal/day)	4.09E-08	9.83E-02	3.48E-01
Waist-to-Hip Ratio	Cigarette years of smoking	1.46E-09	1.34E-03	6.44E-01
Waist-to-Hip Ratio	Physical activity:sports domain	5.55E-10	2.35E-05	2.26E-01
Waist-to-Hip Ratio	Total energy intake (kcal/day)	2.00E-05	2.24E-01	4.35E-01
Waist-to-Hip Ratio	Energy from protein (%kcal/day)	7.11E-07	6.29E-04	7.63E-01
Pulse Pressure	Cigarette years of smoking	3.08E-06	2.50E-03	8.35E-01
Diastolic Blood Pressure	Cigarette years of smoking	5.43E-05	3.52E-04	5.93E-02
Heart Rate	Cigarette years of smoking	1.25E-06	1.37E-02	6.59E-01
Heart Rate	Physical activity:leisure domain	1.01E-04	8.47E-03	2.05E-01
Heart Rate	Physical activity:sports domain	3.74E-06	6.20E-06	4.18E-01
HDL2 Cholesterol	Cigarette years of smoking	1.70E-09	4.02E-02	6.94E-01
HDL2 Cholesterol	Physical activity:leisure domain	2.73E-12	1.32E-01	5.20E-01
HDL2 Cholesterol	Carbohydrate intake (g/day)	1.68E-07	3.42E-01	5.77E-01
HDL2 Cholesterol	Total energy intake (kcal/day)	2.40E-08	2.45E-01	4.50E-01
HDL2 Cholesterol	Energy from protein (%kcal/day)	3.69E-08	7.33E-02	6.54E-01
HDL3 Cholesterol	Alcohol intake (g/week)	3.31E-05	2.45E-02	1.88E-01
HDL3 Cholesterol	Physical activity:sports domain	9.68E-06	1.16E-03	1.52E-01
HDL Cholesterol	Physical activity:sports domain	8.85E-06	9.91E-05	4.71E-02
HDL Cholesterol	Monounsaturated fatty acid intake (g/day)	3.17E-05	4.31E-01	9.37E-01
HDL Cholesterol	Energy from protein (%kcal/day)	4.71E-06	7.86E-02	6.00E-01
Apolipoprotein AI	Polyunsaturated fatty acid intake (g/day)	2.94E-05	2.14E-02	2.13E-01
White Blood Cell Count	Cigarette years of smoking	2.00E-65	2.53E-21	1.25E-08
White Blood Cell Count	Physical activity:leisure domain	7.62E-07	5.19E-01	4.36E-01
White Blood Cell Count	Physical activity:sports domain	9.62E-05	1.72E-01	3.82E-01
White Blood Cell Count	Keys score	7.56E-05	8.37E-01	2.32E-01

Only shown for analyses where the full versus null model comparison was significant. Nested models include the null, G-C only and R-C only models. All analyses are based on data after a rank-based inverse normal transformation.

Table S8. P-values for comparisons between the full model and nested models for the UK Biobank.

Main Trait	Lifestlye Covariate	Null vs. Full	GC vs. Full	RC vs. Full
BMI	Alcohol intake (glass & pint/week)	1.19E-14	0.00E+00	0.00E+00
BMI	MET minutes/week for walking	1.49E-16	4.21E-04	6.82E-02
BMI	MET minutes/week for moderate activity	2.05E-29	6.34E-13	8.26E-01
BMI	MET minutes/week for vigorous activity	1.01E-46	8.91E-20	4.74E-01
BMI	Summed MET minutes/week for all activity	5.51E-47	3.16E-15	2.71E-01
BMI	estimated saturated fat intake	6.44E-03	6.37E-01	3.85E-01
Diastolic Blood Pressure	Pack years adult smoking as proportion of life span exposed to smoking	3.03E-04	1.75E-04	1.08E-02
Pulse Pressure	Pack years adult smoking as proportion of life span exposed to smoking	7.13E-06	8.66E-02	4.10E-01
Heart Rate	Pack years adult smoking as proportion of life span exposed to smoking	3.44E-38	5.01E-05	3.92E-01
Heart Rate	MET minutes/week for walking	3.81E-02	6.38E-02	6.96E-01
Heart Rate	Summed MET minutes/week for all activity	3.62E-02	1.05E-01	9.20E-01
Waist-to-Hip Ratio	Pack years adult smoking as proportion of life span exposed to smoking	5.35E-87	6.21E-15	7.46E-01
Waist-to-Hip Ratio	MET minutes/week for moderate activity	9.00E-04	2.07E-01	2.21E-01
Waist-to-Hip Ratio	MET minutes/week for vigorous activity	1.13E-04	2.46E-01	3.12E-01
Waist-to-Hip Ratio	Summed MET minutes/week for all activity	2.23E-04	4.45E-01	2.65E-01
Waist-to-Hip Ratio	estimated total energy intake	2.40E-02	1.81E-02	1.18E-01
White Blood Cell Count	Pack years adult smoking as proportion of life span exposed to smoking	1.25E-138	3.58E-21	6.04E-01
HDL Cholesterol	MET minutes/week for moderate activity	8.01E-08	2.77E-01	3.27E-01
HDL Cholesterol	MET minutes/week for vigorous activity	4.37E-04	5.01E-01	7.39E-01
HDL Cholesterol	Summed MET minutes/week for all activity	5.91E-06	7.65E-01	6.91E-01

Only shown for analyses where the full versus null model comparison was significant. Nested models include the null, G-C only and R-C only models. All analyses are based on data after a rank-based inverse normal transformation.

Table S9. Genomic relationship within and between top and bottom groups, stratified by G-C interaction estimate, relative to the grand mean genomic relationship.

Main Trait	Lifestyle	Within Top Group		Within Bottom Group		Between Top & Bottom Groups	
		Δ%[1]	*p-value* [2]	Δ%	*p-value*	Δ%	*p-value*
HDL Cholesterol	Physical activity:sports domain	63.4	9.47E-72	77.0	1.46E-105	-69.5	3.44E-165
HDL3 Cholesterol	Physical activity:sports domain	64.3	2.44E-73	67.4	3.47E-81	-66.0	2.72E-148
White Blood Cell Count	Physical activity:sports domain	62.4	2.81E-69	61.4	5.66E-67	-64.0	3.12E-139
HDL2 Cholesterol	Energy from prot1 (%kcal/day)	84.3	2.20E-125	87.8	4.48E-136	-82.6	4.90E-233
White Blood Cell Count	Physical activity:leisure domain	67.3	5.40E-81	66.1	2.03E-77	-65.2	4.46E-145
BMI	Saturated fatty acid intake (g/day)	66.1	4.45E-80	94.0	1.40E-148	-76.3	5.44E-201
HDL3 Cholesterol	Alcohol intake (g/week)	55.1	1.18E-54	57.0	1.71E-58	-56.5	1.72E-109
Heart Rate	Physical activity:sports domain	55.2	1.72E-54	53.2	6.04E-52	-54.1	1.72E-101

1. Δ% = (group mean - grand mean)/grand mean x 100%; 2. P-values are for two-sided independent t-tests that compare group means with the grand mean

Figure S1. Skewness (top) and kurtosis (bottom) of cardiovascular traits from the atherosclerosis risk in communities study (ARIC) and UK biobank (UKBB).

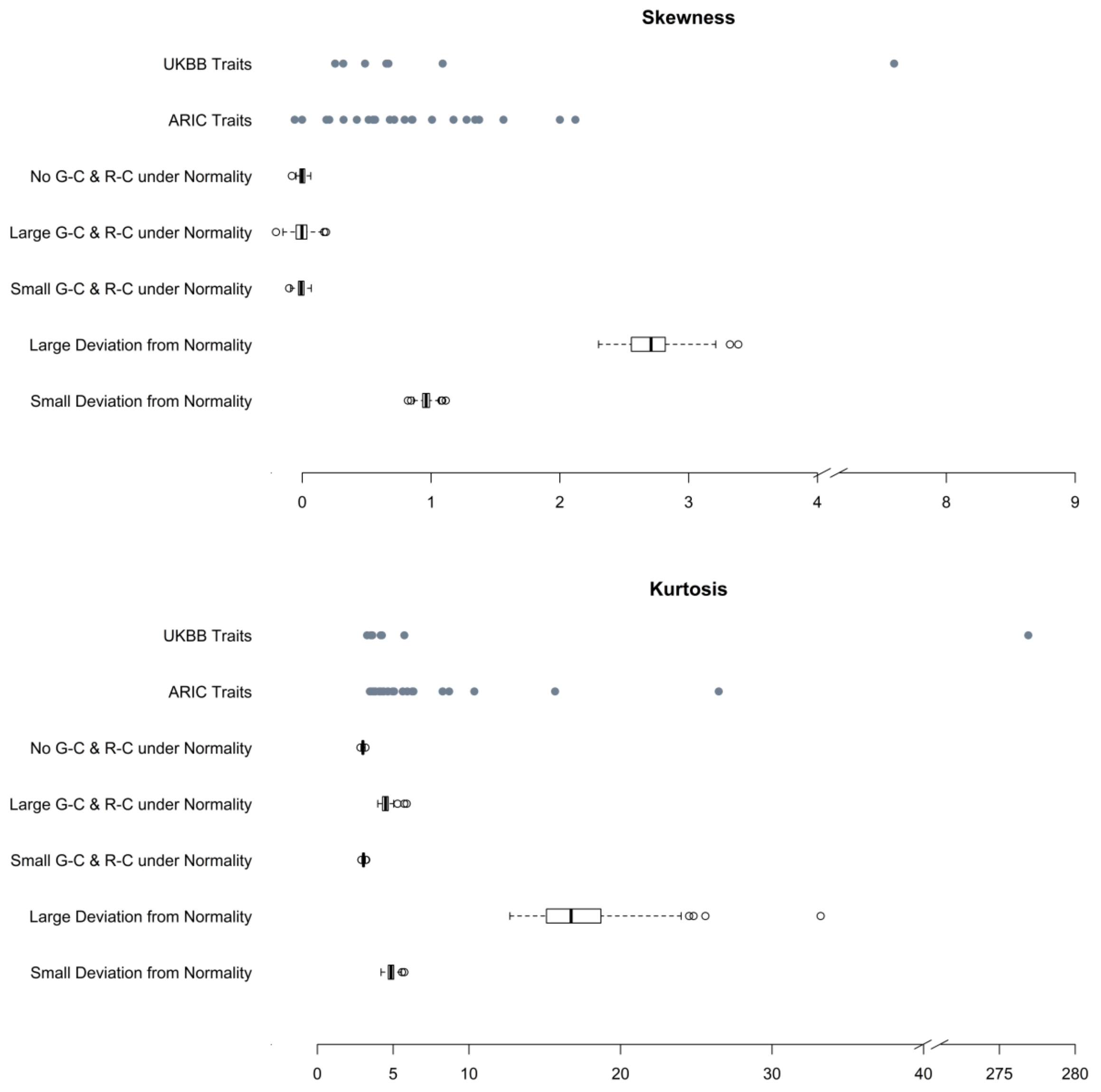

In each panel, categories along the y axis from the top to bottom are traits from the UK biobank, traits from the ARIC study, traits (100 replicates) simulated from the null model, from the full model with large and small effect sizes, and from the full model with non-normal residuals that result in large and small phenotypic deviations from normality. The last five categories are included as references to indicate expected skewness and kurtosis when the model assumption of normality is and is not met.

Figure S2. Sampling distributions of parameter estimates from the full model.

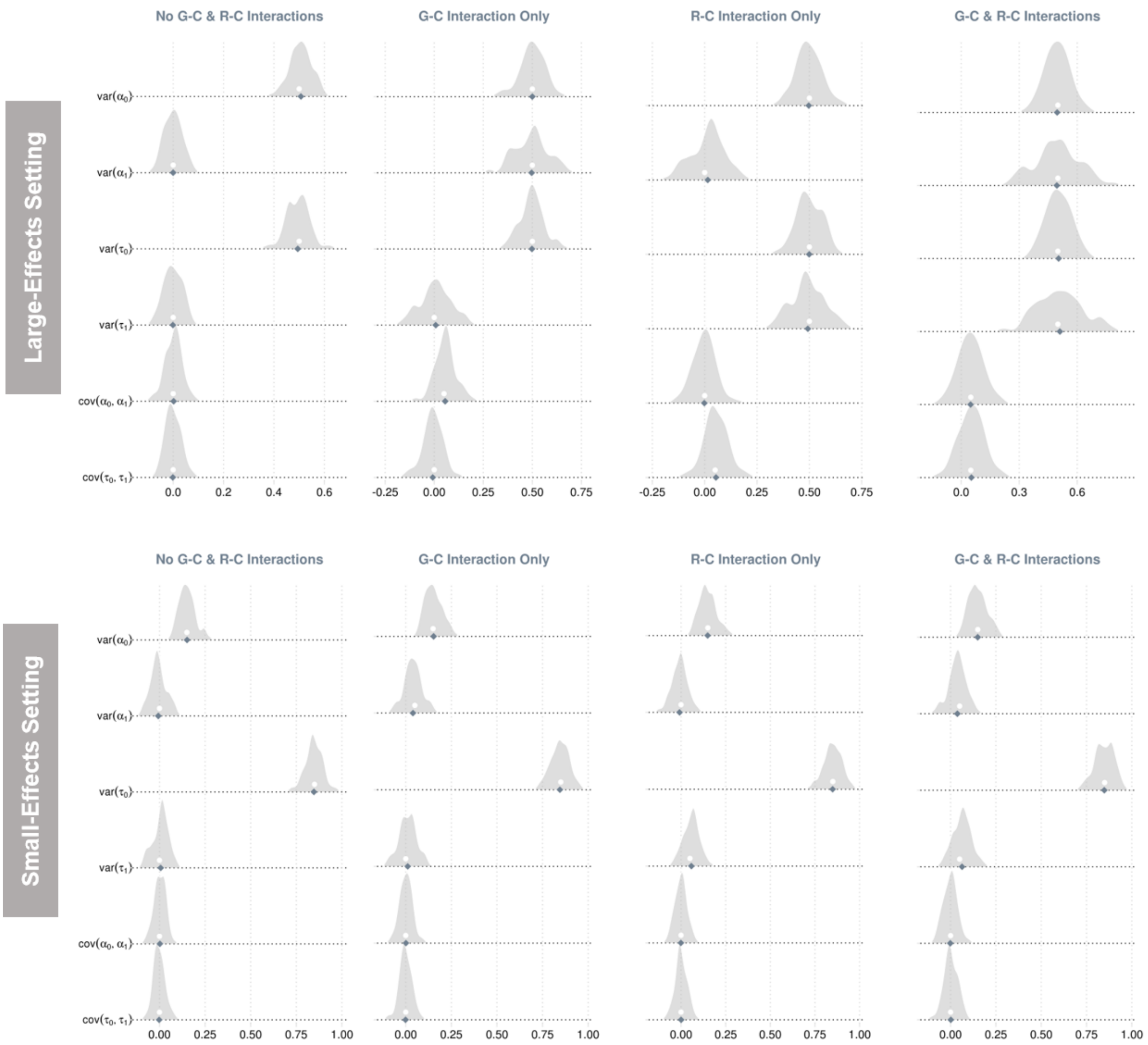

Data for a main trait and a covariate were simulated using 4 multivariate reaction norm models under 2 parameter settings for each model, which together gave arise to 8 combinations of simulation scenarios. The four models were no Genotype-Covariate (G-C) and Residual-Covariate (R-C) interactions (i.e., a null model), G-C interaction only (i.e., a G-C model), R-C interaction only (i.e., a R-C model), and both G-C and R-C interactions (i.e., a full model). The two parameter settings were large and small effect sizes in terms of heritability, G-C and R-C interactions. Each simulation was repeated 100 times, resulting in 100 replicates of simulated data under each scenario. Parameter estimates were obtained from fitting the full model. Shown distributions are for model parameters (i.e., variance & covariance terms) pertaining to the main trait only. True parameter values are shown in dots and means of sampling distributions in diamonds.

Figure S3. Impact of rank-based inverse normal transformation on parameter estimates when the normality assumption is violated.

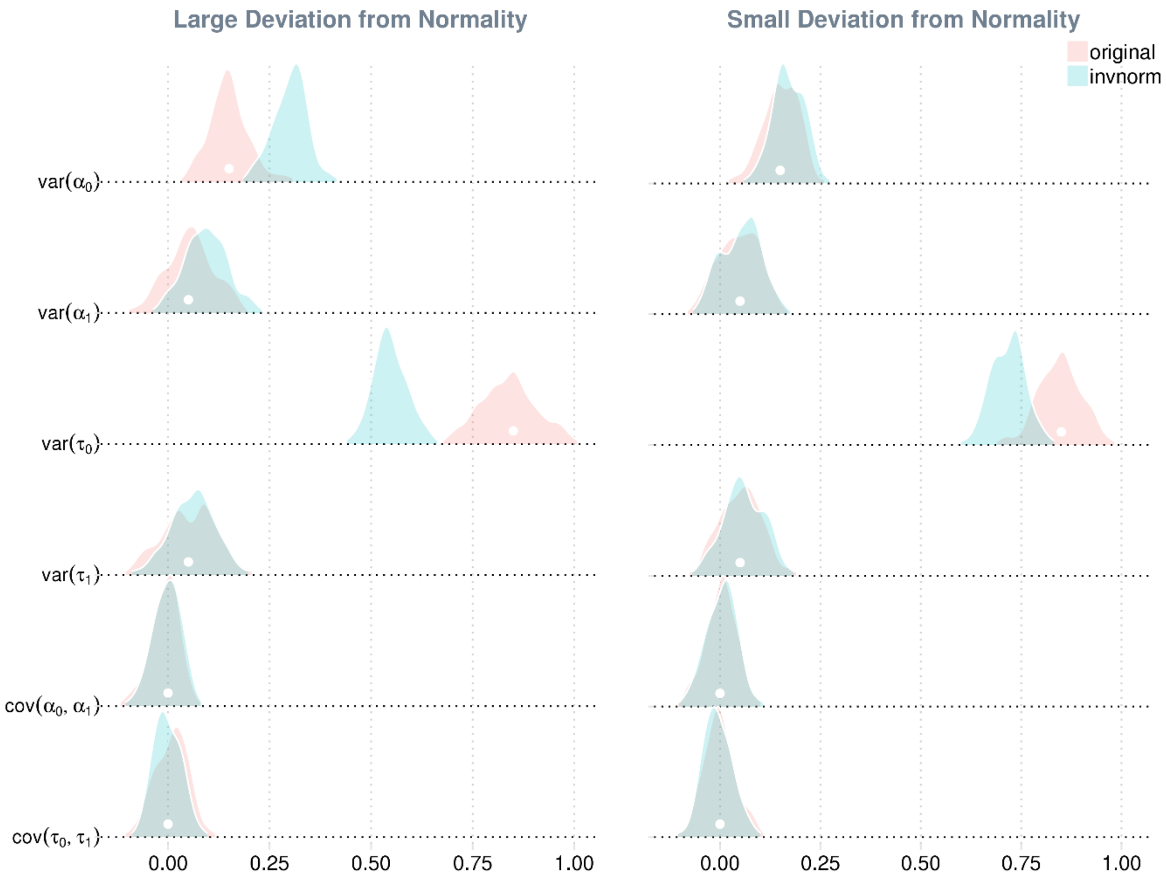

Data for a main trait and a covariate were simulated using a multivariate reaction norm model that included both Genotype-Covariate and Residual-Covariate interactions (i.e., a full model) under two parameter settings, where residuals of the main trait were drawn from distributions that deviated from a normal distribution to different degrees (i.e., small vs. large deviation from normality). Each simulation was repeated 100 times, resulting in 100 replicates of simulated data for each setting. Parameter estimates were obtained from fitting a full model—that assumes normality of all random effects including residuals—to the simulated data before and after a rank-based inverse normal distribution ('original' vs. 'invnorm'). Shown distributions are for model parameters (i.e., variance & covariance terms) pertaining to the main trait only. True parameter values are shown in dots.

Figure S4. Impact of rank-based inverse normal transformation on parameter estimates when normality assumption is met.

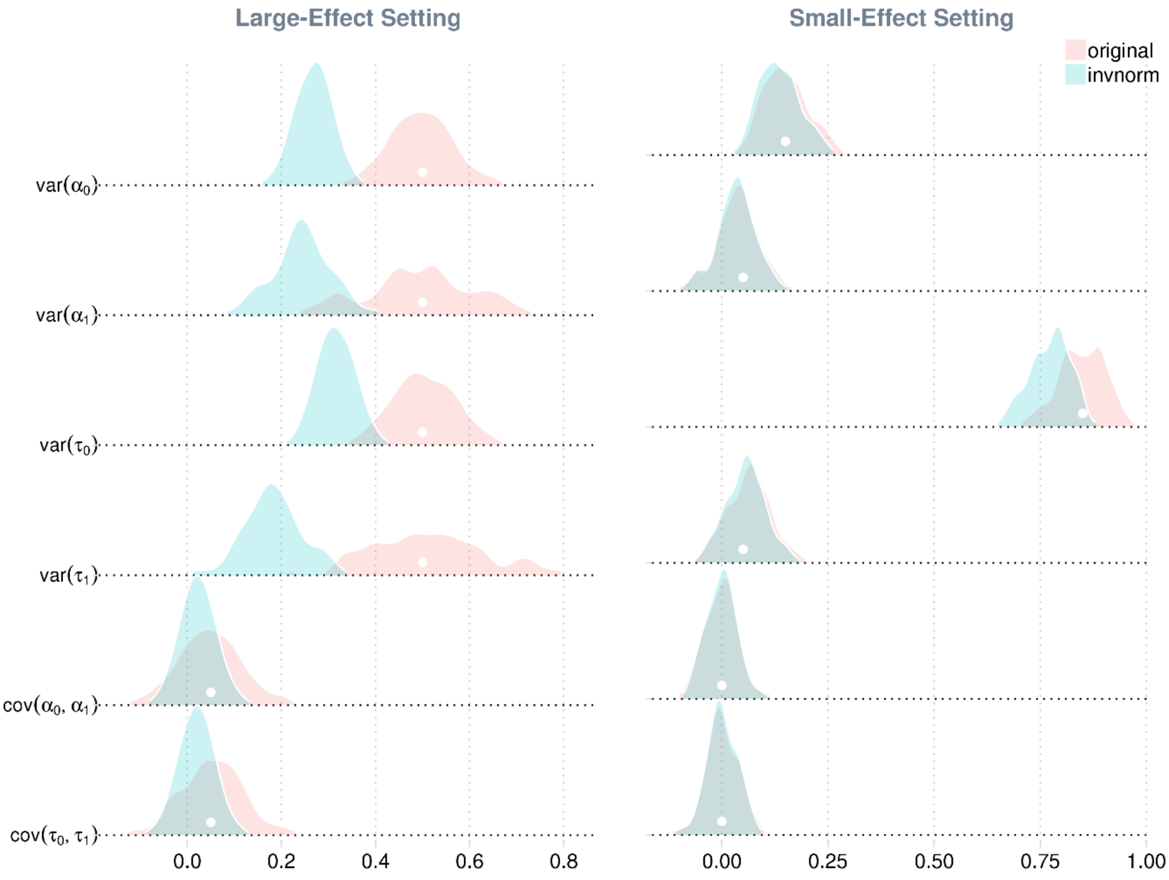

Data for a main trait and a covariate were simulated using a multivariate reaction norm model that included both Genotype-Covariate (G-C) and Residual-Covariate (R-C) interactions (i.e., a full model) under two parameter settings that varied in effect sizes in terms of heritability, G-C and R-C interactions. In each setting, all random effects were drawn from normal distributions. Each simulation was repeated 100 times, resulting in 100 replicates of simulated data for each setting. Parameter estimates were obtained from fitting a full model—that assumes normality of random effects—to the simulated data before and after a rank-based inverse normal distribution ('original' vs. 'invnorm'). Shown distributions are for model parameters (i.e., variance & covariance terms) pertaining to the main trait only. True parameter values are shown in dots.

Figure S5. Sampling distributions of parameter estimates for four multivariate reaction norm models under four simulation scenarios for the small-effects parameter setting.

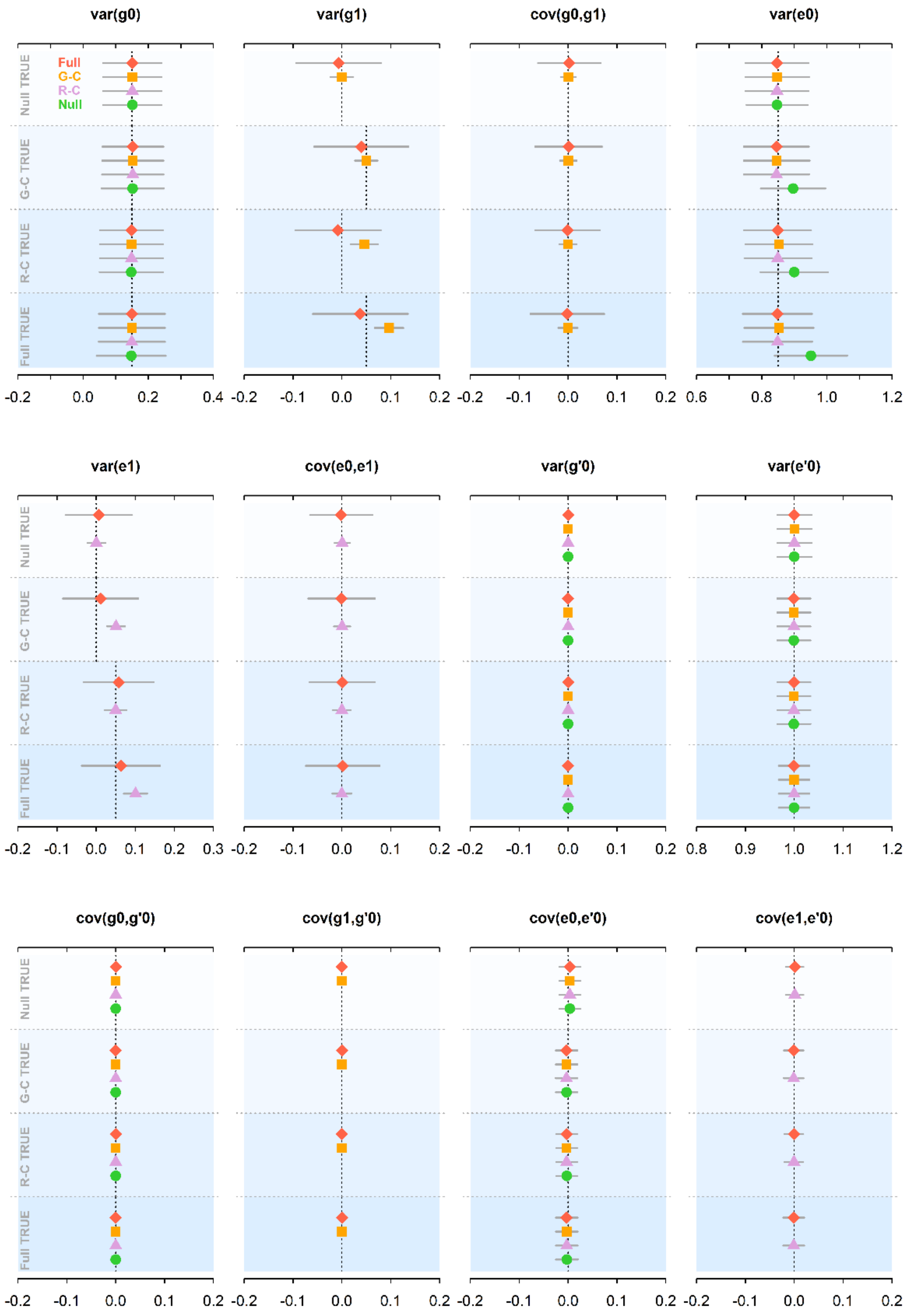

The four scenarios are no Genotype-Covariate (G-C) and Residual-Covariate (R-C) interactions (i.e., a null model), G-C interaction only (i.e., a G-C model), R-C interaction only (i.e., a R-C model), and both G-C and R-C interactions (i.e., a full model). There are 100 replicates under each scenario, with the true value of each parameter being indicated by a vertical dashed line.

Figure S6. Skewness (left panel) and kurtosis (right) of cardiovascular traits from the ARIC dataset by survivorship of rank-based inverse normal transformation.

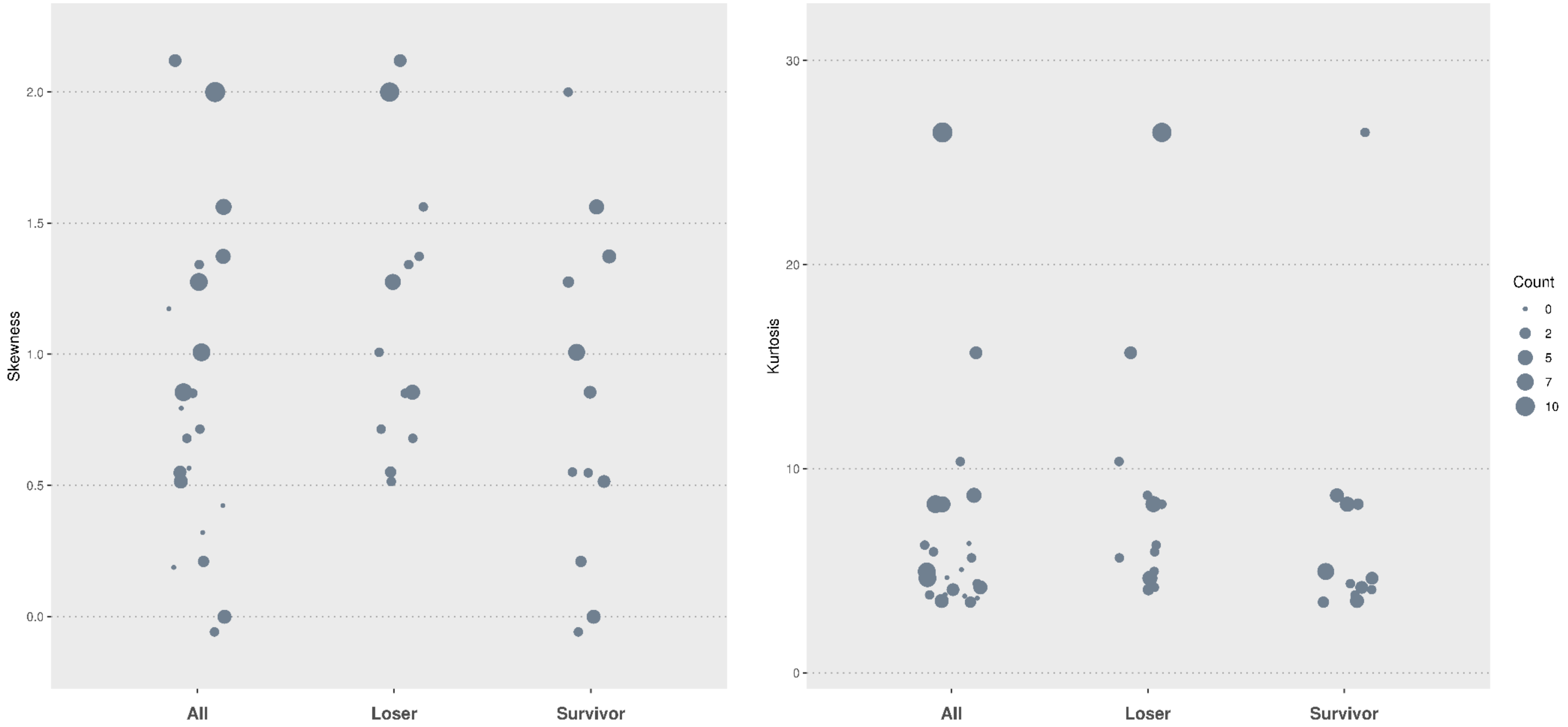

Categories from left to right in each panel are all traits (labelled as “all”), trait that lost one or more signals after a rank-based inverse normal transformation (“loser”), and traits that still had one or more signals after the transformation (“survivor”). Point size is proportional to the count of signals. To reduce overlaps, points are jittered randomly in the horizontal direction. Note that a trait can be both a loser and a survivor.

Figure S7. Estimated genetic covariance with respect to lifestyle covariate.

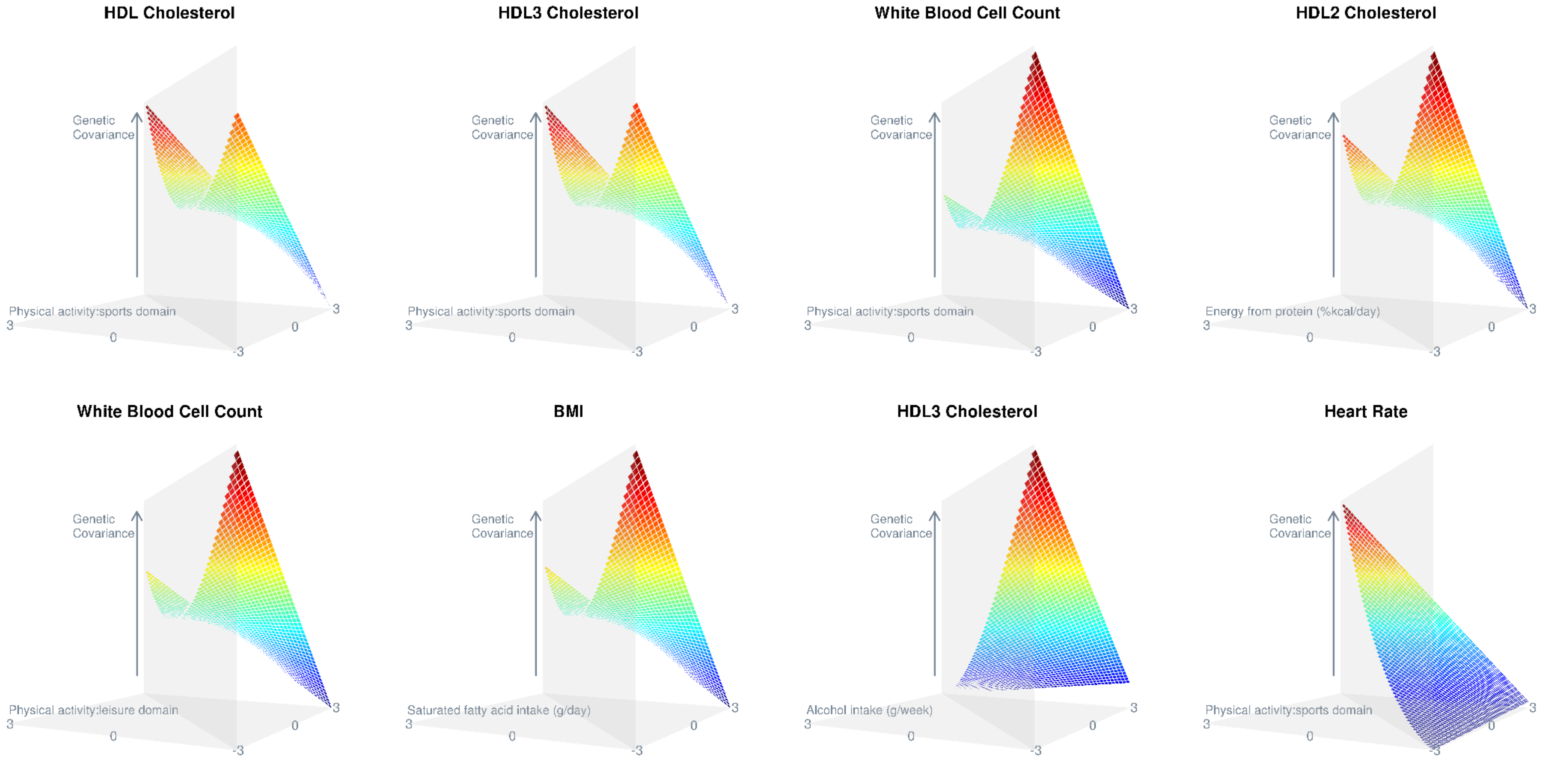

Both x and y coordinates of each plot cover three standard deviations from the mean of a given lifestyle covariate. The horizontal plane thus represents pairwise combinations of lifestyle covariate values. The corresponding genetic covariance matrix of these combinations, estimated from the full model, is shown as the surface in each plot. The lower triangular part of each matrix, which is identical as the upper triangular part, is removed for simplicity. Diagonal entries of each matrix, shown as the intersection of the surface with the diagonal plane, are estimated genetic variances. Only traits with the first eight largest variance estimates of Genotype-Covariate interaction (see Figure 2 left) are shown. Arrows point to higher values.

Figure S8. Estimated residual covariance with respect to lifestyle covariate.

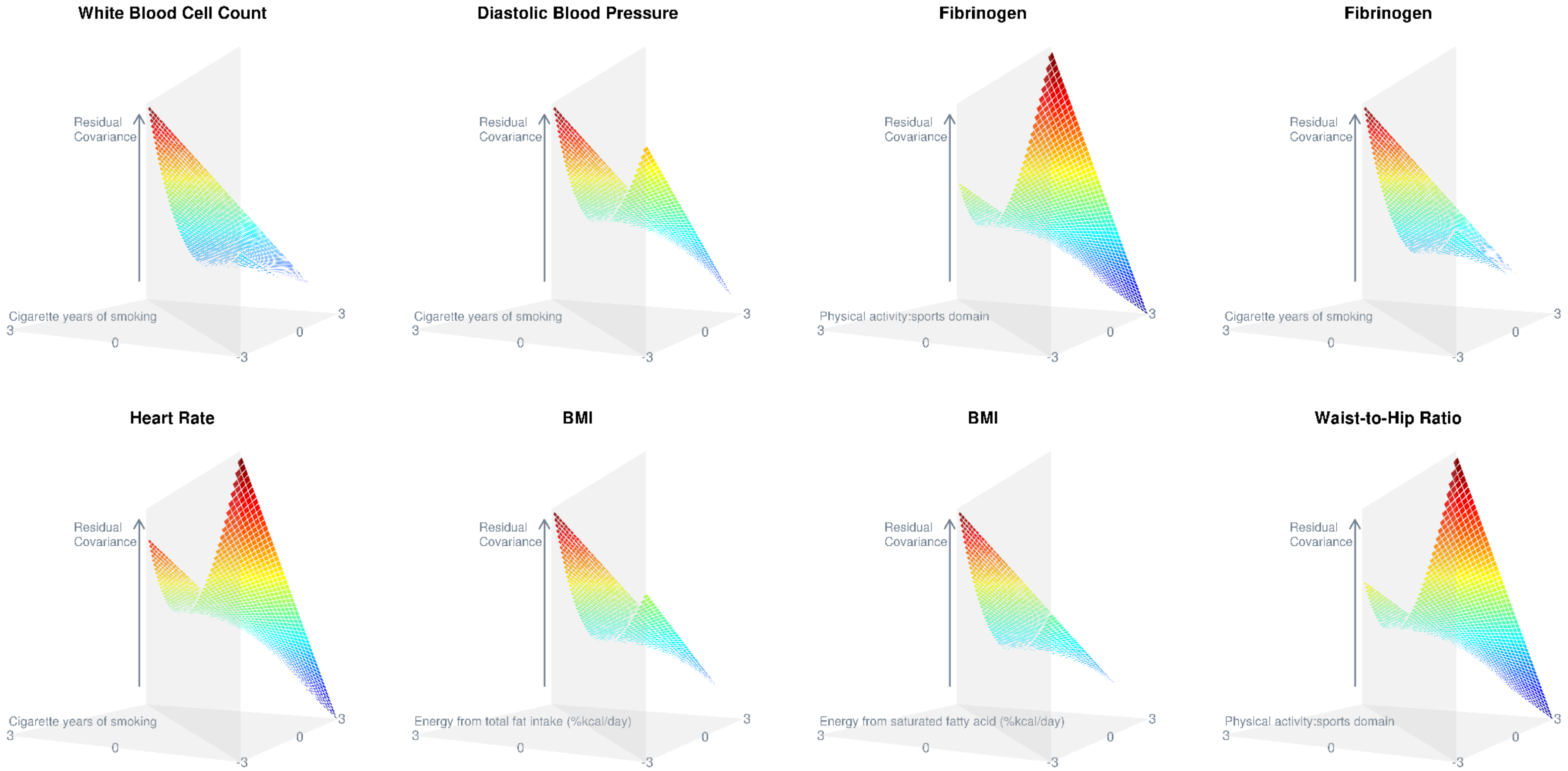

Both x and y coordinates of each plot cover three standard deviations from the mean of a given lifestyle covariate. The horizontal plane thus represents pairwise combinations of lifestyle covariate values. The corresponding residual covariance matrix of these combinations, estimated from the full model, is shown as the surface in each plot. The lower triangular part of each matrix, which is identical as the upper triangular part, is removed for simplicity. Diagonal entries of each matrix, shown as the intersection of the surface with the diagonal plane, are estimated residual variances. Only traits with the first eight largest variance estimates of Residual-Covariate interaction (see Figure 2 right) are shown. Arrows point to higher values.
